# Supplementary material for: Room-temperature quantum interference in single perovskite quantum dot junctions
Source: Nat Commun. 2019 Nov 29;10:5458. doi: 10.1038/s41467-019-13389-7 (PMC6884635; doi:10.1038/s41467-019-13389-7)
Supplement: Supplementary file 1 — Supplementary Information [file 41467_2019_13389_MOESM1_ESM.pdf]

Supplementary Information for

Room-temperature Quantum Interference in Perovskite Quantum Dot Junctions

Zheng et al.

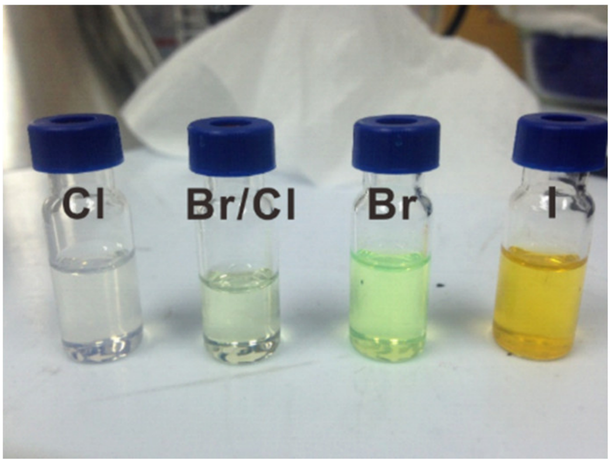

Supplementary Figure 1 | Perovskite quantum dots (QDs) solution. a, The picture of $MAPbCl_3$, $MAPbBr_{2.15}Cl_{0.85}$, $MAPbBr_3$ and $MAPbI_3$ perovskite QDs (from left to right).

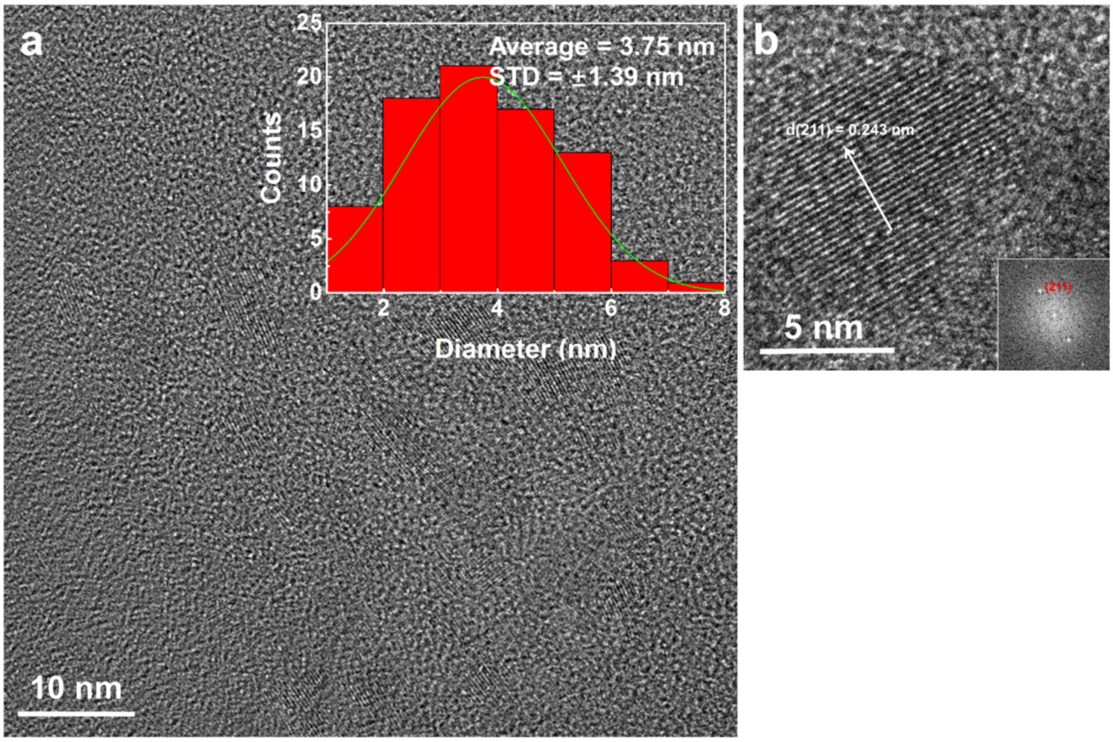

Supplementary Figure 2 | The image of transmission electron microscopy. a, The relative diameter distribution for $MAPbBr_3$ perovskite QDs. **b,** The high-resolution transmission electron microscopy (HRTEM) image.

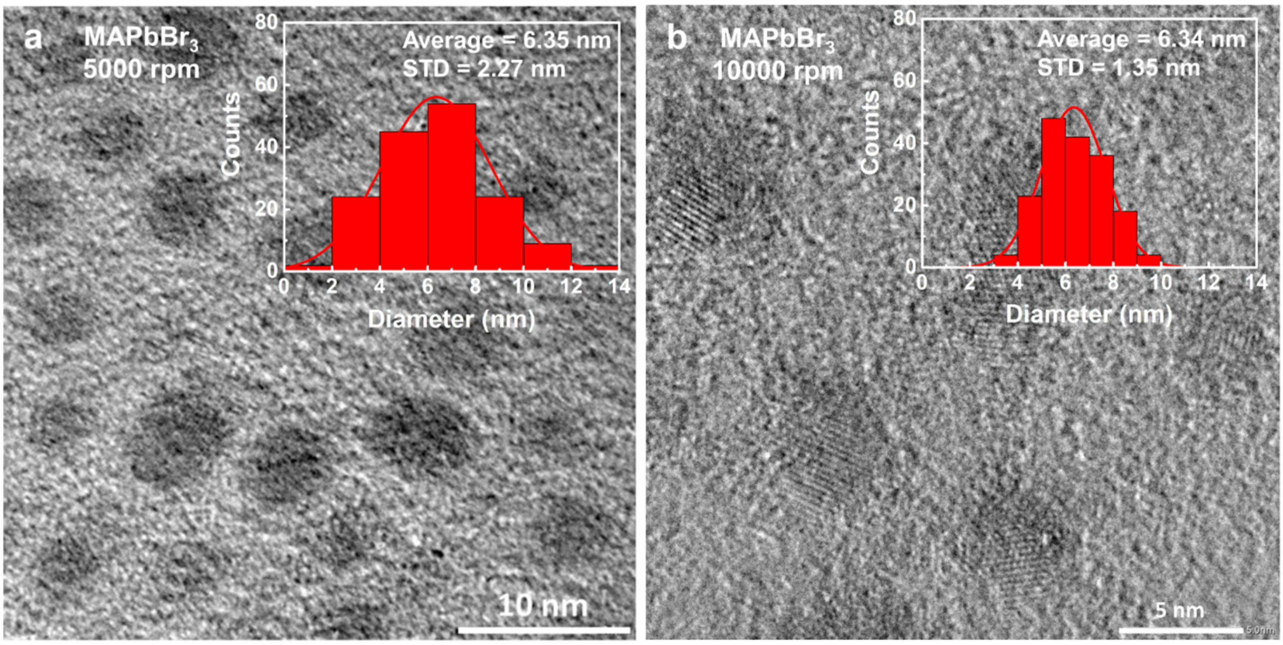

Supplementary Figure 3 | The image of transmission electron microscopy for $MAPbBr_3$ perovskite QDs with different centrifugal speeds. a, The HRTEM image for $MAPbBr_3$ perovskite QDs with the centrifugal speed of 5000 rpm. **b,** The HRTEM image for $MAPbBr_3$ perovskite QDs with the centrifugal speed of 10000 rpm.

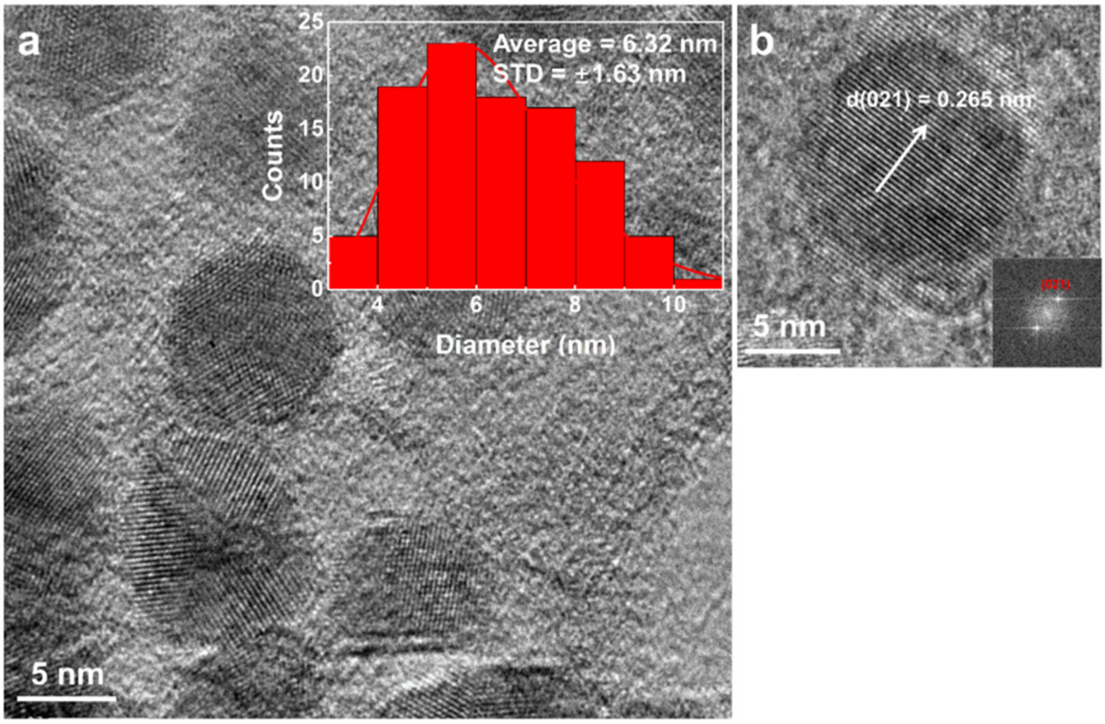

Supplementary Figure 4 | The image of transmission electron microscopy. a, The relative diameter distribution for $MAPbBr_{2.15}Cl_{0.85}$ perovskite QDs. **b,** The high-resolution transmission electron microscopy (HRTEM) image.

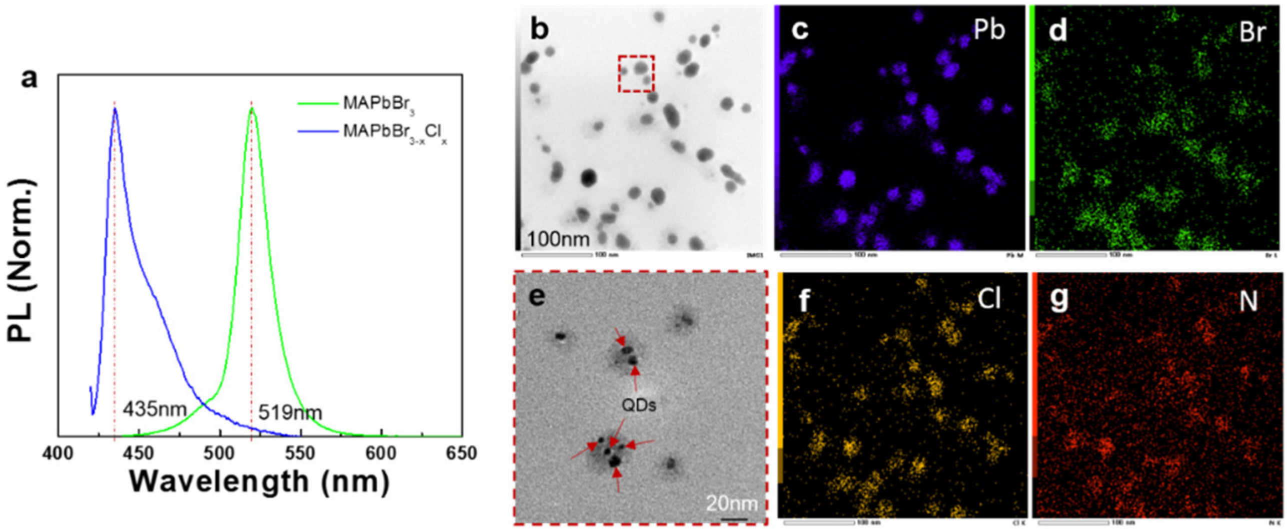

Supplementary Figure 5 | The Photoluminescence of perovskite QDs. a, Photoluminescence (PL) of $MAPbBr_3$ and $MAPbBr_{2.15}Cl_{0.85}$ QDs solution in Toluene. **b, e,** TEM images of $MAPbBr_{2.15}Cl_{0.85}$ QDs. **c, d, f, g,** EDS mapping of $MAPbBr_{2.15}Cl_{0.85}$ QDs.

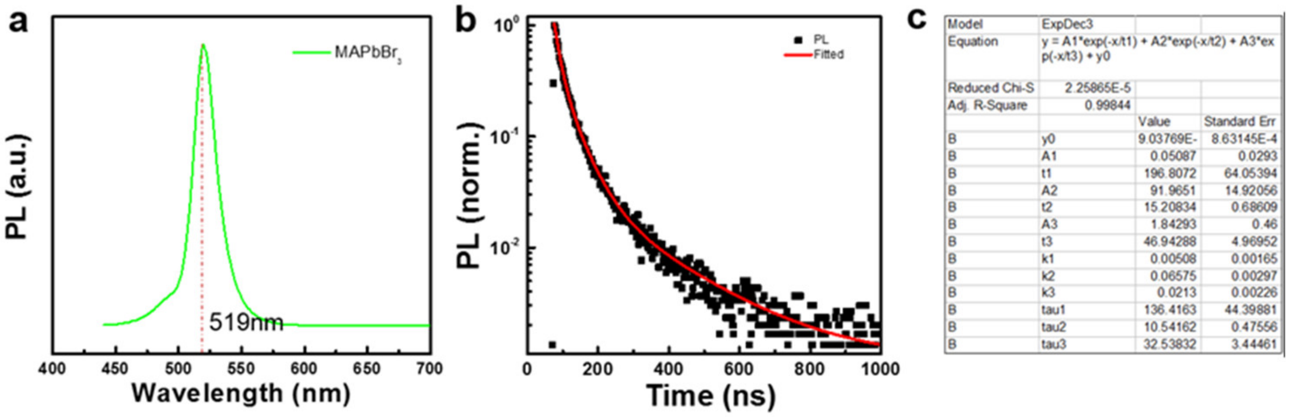

Model	ExpDec3		
Equation	y = A1*exp(-x/t1) + A2*exp(-x/t2) + A3*exp(-x/t3) + y0		
Reduced Chi-S	2.25865E-5		
Adj. R-Square	0.99844		
		Value	Standard Err
B	y0	9.03769E-	8.63145E-4
B	A1	0.05087	0.0293
B	t1	196.8072	64.05394
B	A2	91.9651	14.92056
B	t2	15.20834	0.68609
B	A3	1.84293	0.46
B	t3	46.94288	4.96952
B	k1	0.00508	0.00165
B	k2	0.06575	0.00297
B	k3	0.0213	0.00226
B	tau1	136.4163	44.39881
B	tau2	10.54162	0.47556
B	tau3	32.53832	3.44461

Supplementary Figure 6 | The Photoluminescence and time-resolved photoluminescence of perovskite QDs. a, PL of $MAPbBr_3$ QDs solution in Toluene. **b,** Time-resolved PL (TRPL) of $MAPbBr_3$ quantum dots (QDs) in Toluene. **c,** the fitted results of TRPL.

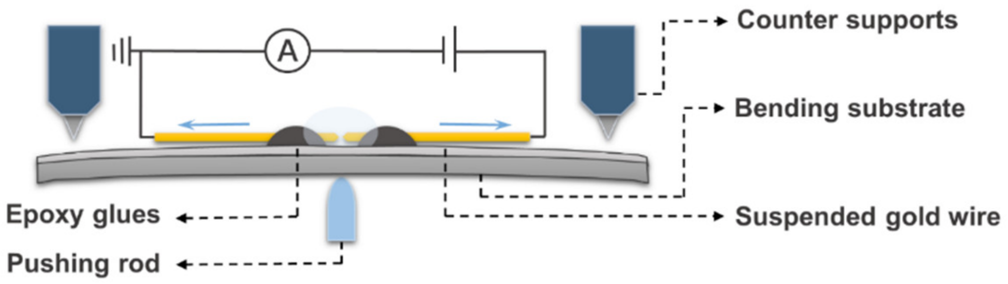

Supplementary Figure 7 | The schematic of the MCBJ experimental principle.

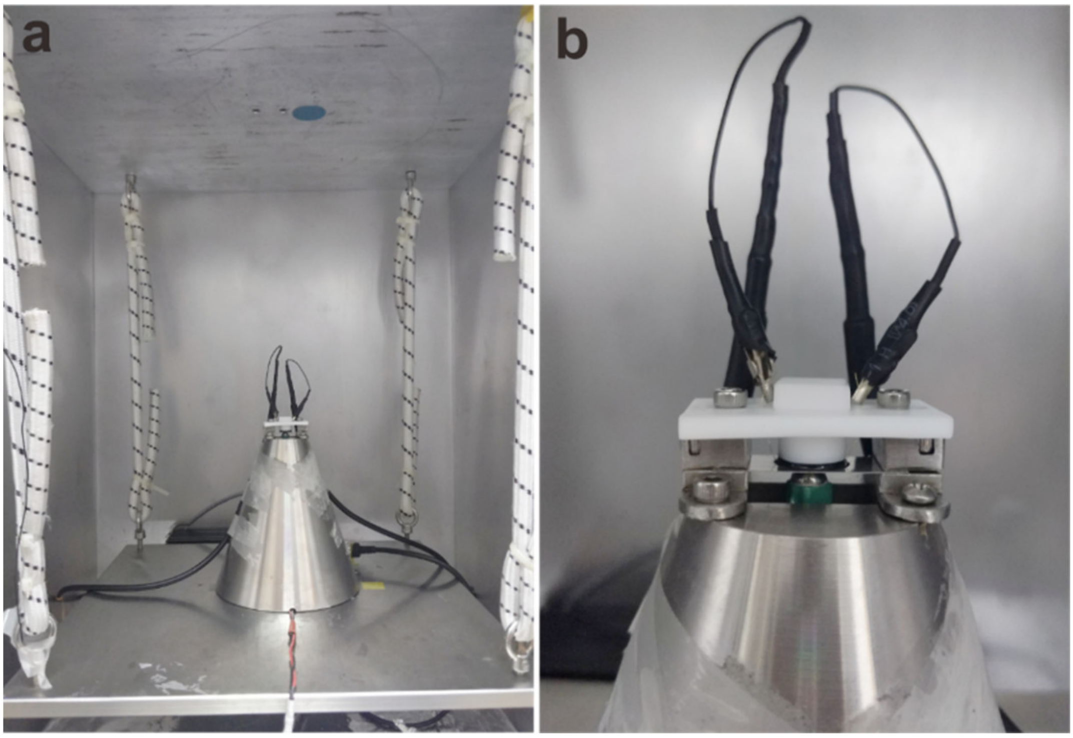

Supplementary Figure 8 | The MCBJ setup. a, The MCBJ setup for the whole view. **b,** the MCBJ setup for enlarged view of the chip with liquid cell.

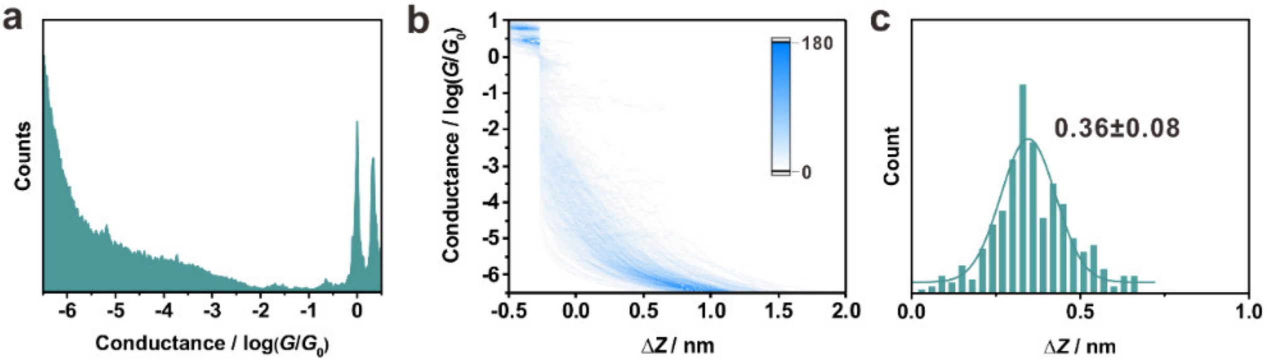

Supplementary Figure 9 | The control experiments of pure solvent and the calibration of displacement. a, The 1D conductance histogram of a blank experiment for pure solvent. **b,** 2D conductance versus relative distance (Δz) histogram. **c,** the relative displacement distribution histogram determined from the conductance range between $10^{-3.5}$ G_0 to $10^{-5.5}$ G_0. Clean background suggested the solution environment is clean and no molecular junctions are formed during the measurement.

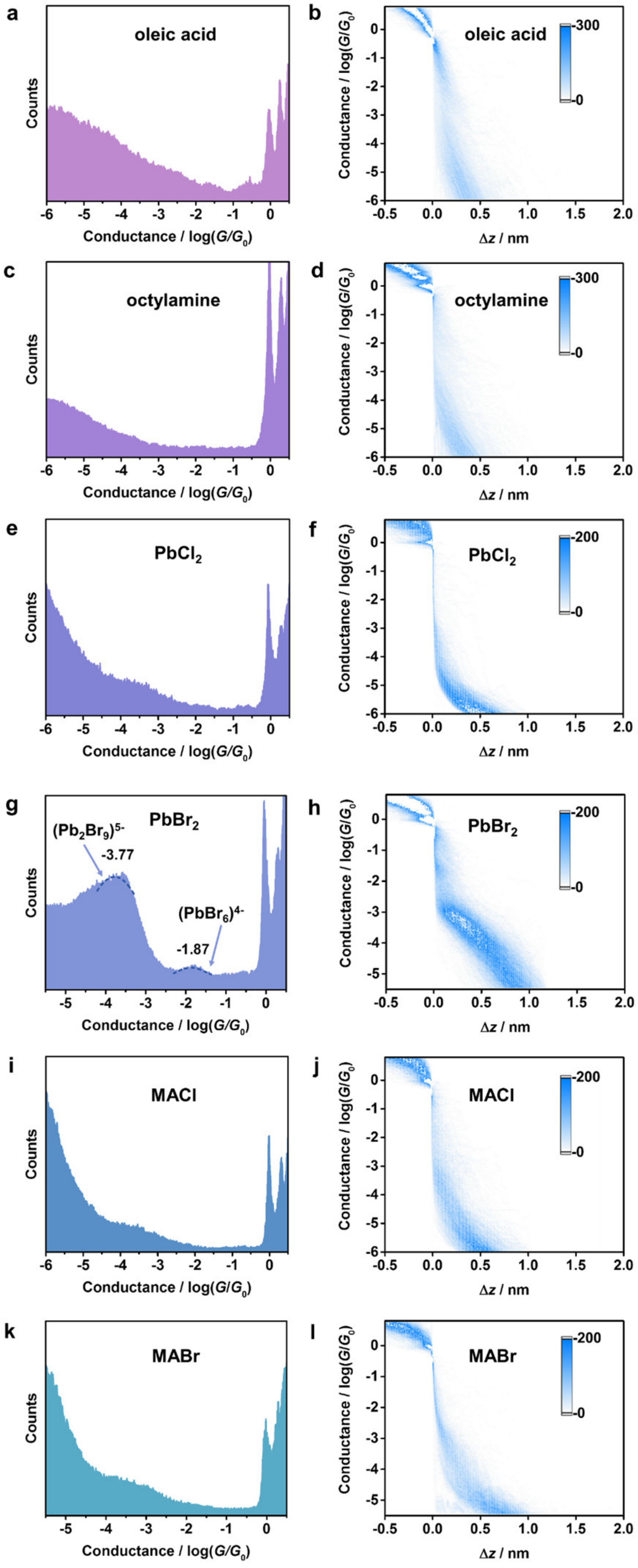
a
oleic acid
Counts
Conductance / log(G/G0)
b
oleic acid
Conductance / log(G/G0)
Δz / nm
300
0
c
octylamine
d
octylamine
e
$PbCl_2$
f
$PbCl_2$
200
g
$PbBr_2$
$(Pb_2Br_9)^{5-}$
-3.77
$(PbBr_6)^{4-}$
-1.87
h
$PbBr_2$
i
MACl
j
MACl
k
MABr
l
MABr

Supplementary Figure 10 | The MCBJ measurements of ligands and synthetic raw materials. a, 1D conductance histograms for oleic acid. **b,** The 2D conductance versus relative distance (Δz) histogram for oleic acid. **c,** 1D conductance histograms for octylamine. **d,** The 2D conductance versus relative distance (Δz) histogram for octylamine. **e,** 1D conductance histograms for $PbCl_2$. **f,** The 2D conductance versus relative distance (Δz) histogram for $PbCl_2$. **g,** 1D conductance histograms for $PbBr_2$. **h,** The 2D conductance versus relative distance (Δz) histogram for $PbBr_2$. **i,** 1D conductance histograms for MACl. **j,** The 2D conductance versus relative distance (Δz) histogram for MACl. **k,** 1D conductance histograms for MABr. **l,** The 2D conductance versus relative distance (Δz) histogram for MABr.

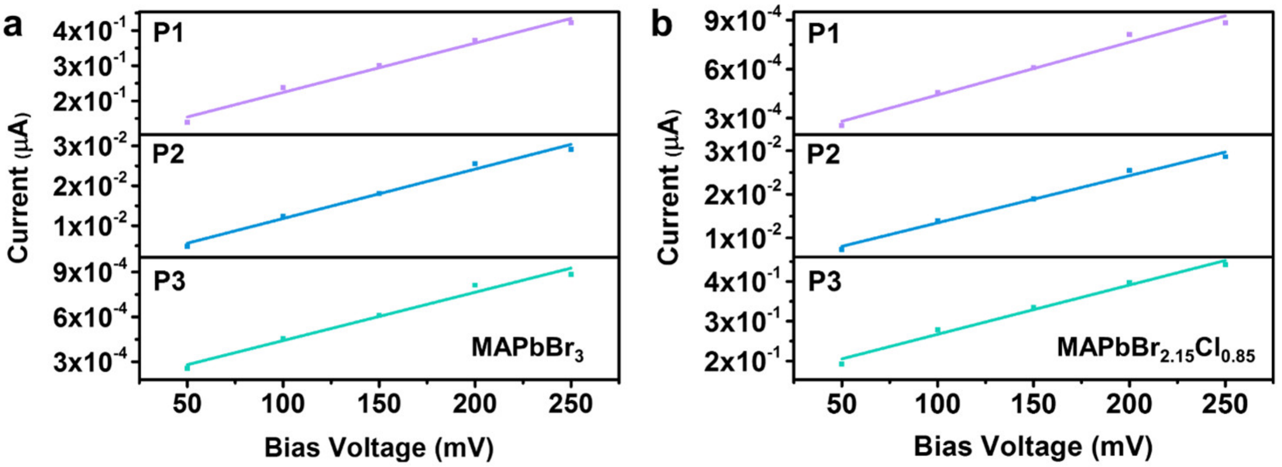

Supplementary Figure 11 | The *I-V* characteristic curves of perovskite QDs. a, the I-V characteristics of $MAPbBr_3$ determined from conductance histograms at different bias voltages. **b,** the I-V characteristics of $MAPbBr_{2.15}Cl_{0.85}$ determined from conductance histograms at different bias voltages.

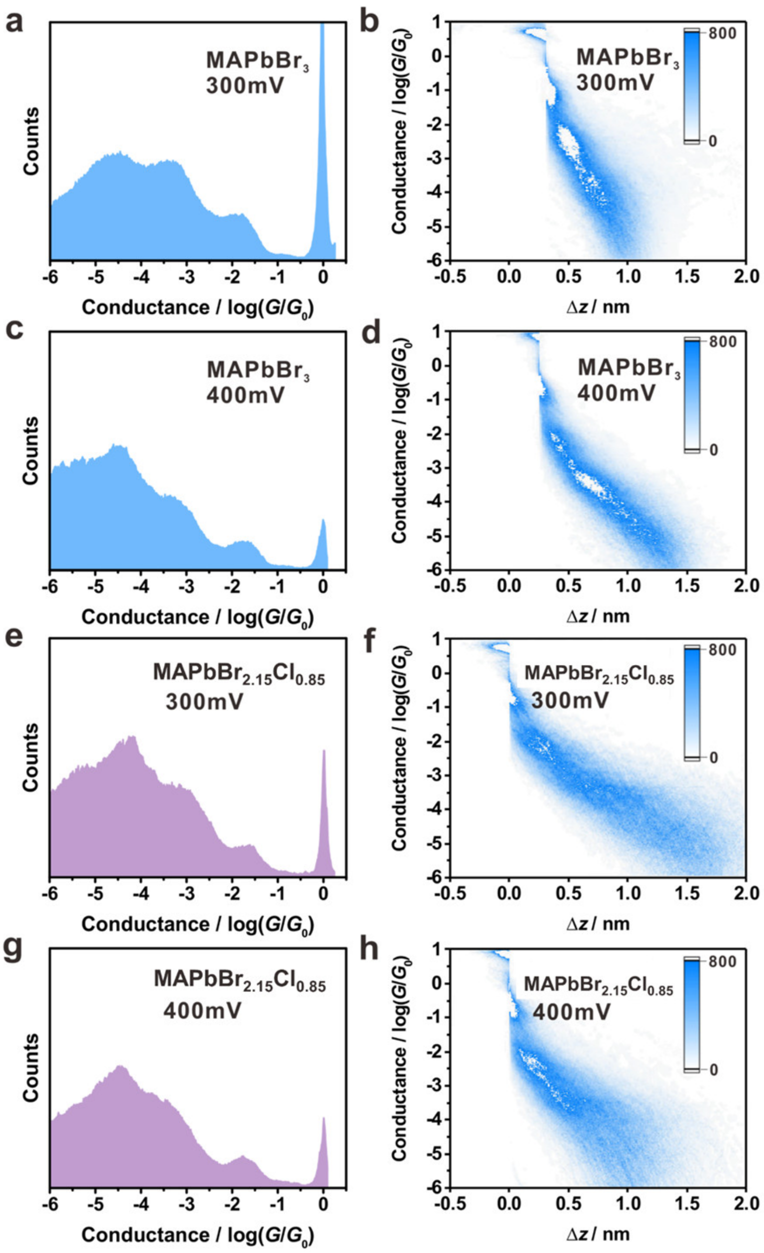

Supplementary Figure 12 | The MCBJ measurement of $MAPbBr_3$ and $MAPbBr_{2.15}Cl_{0.85}$ at bias 300mV and 400mV. a, 1D conductance histograms without data selection for perovskite QDs $MAPbBr_3$ (300 mV). **b,** The 2D conductance versus relative distance (Δz) histogram for perovskite QDs $MAPbBr_3$ (300 mV). **c,** 1D conductance histograms without data selection for perovskite QDs $MAPbBr_3$ (400 mV). **d,** The 2D conductance versus relative distance (Δz) histogram for perovskite QDs $MAPbBr_3$ (400 mV). **e,** 1D conductance histograms without data selection for perovskite QDs $MAPbBr_{2.15}Cl_{0.85}$ (300 mV). **f,** The 2D conductance versus relative distance (Δz) histogram for perovskite QDs $MAPbBr_{2.15}Cl_{0.85}$ (300 mV). **g,** 1D conductance histograms without data selection for perovskite QDs $MAPbBr_{2.15}Cl_{0.85}$ (400 mV). **h,** The 2D conductance versus relative distance (Δz) histogram for perovskite QDs $MAPbBr_{2.15}Cl_{0.85}$ (400 mV).

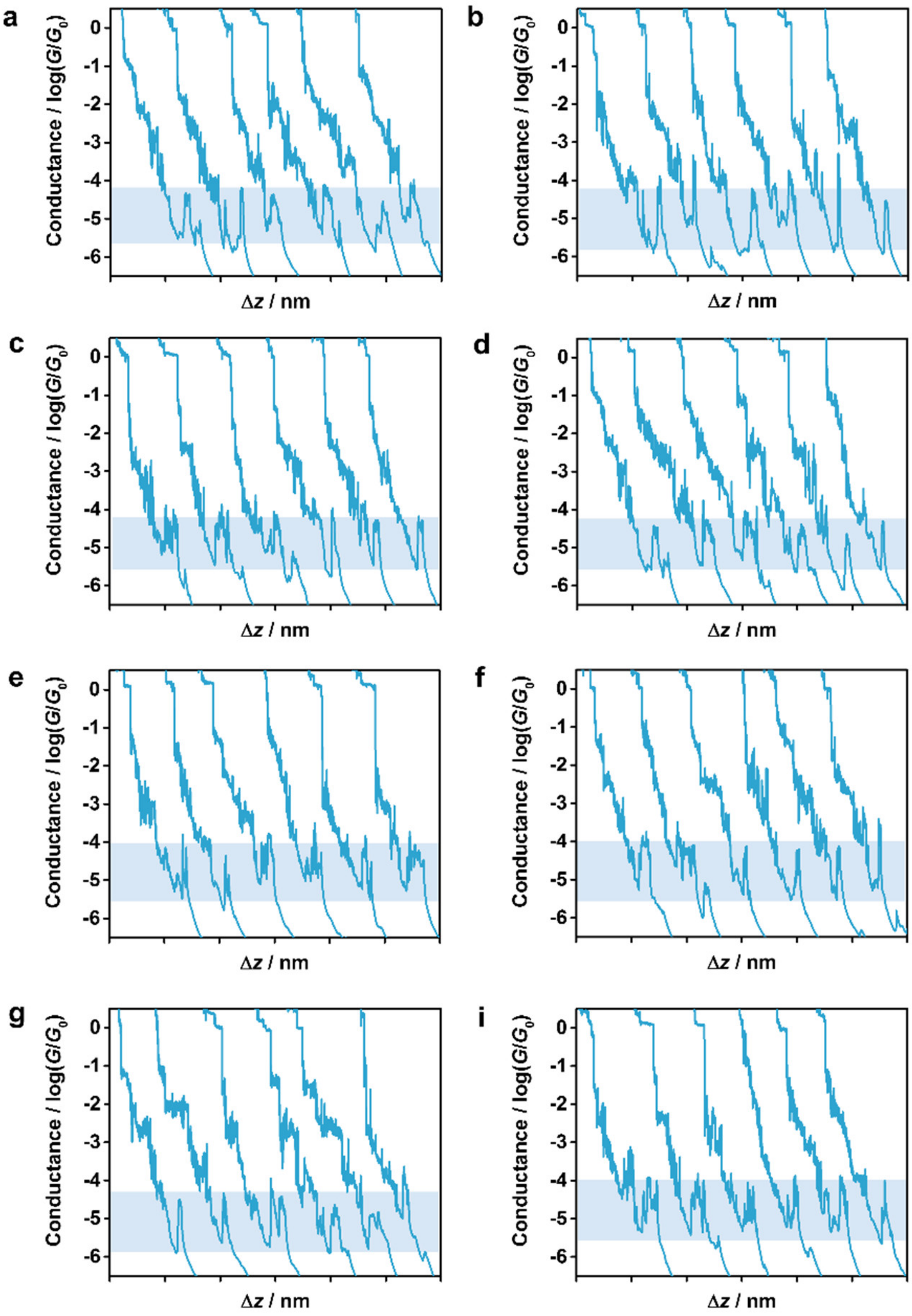

Supplementary Figure 13 | The individual "jump" conductance-distance traces of $MAPbBr_3$ perovskite QDs.

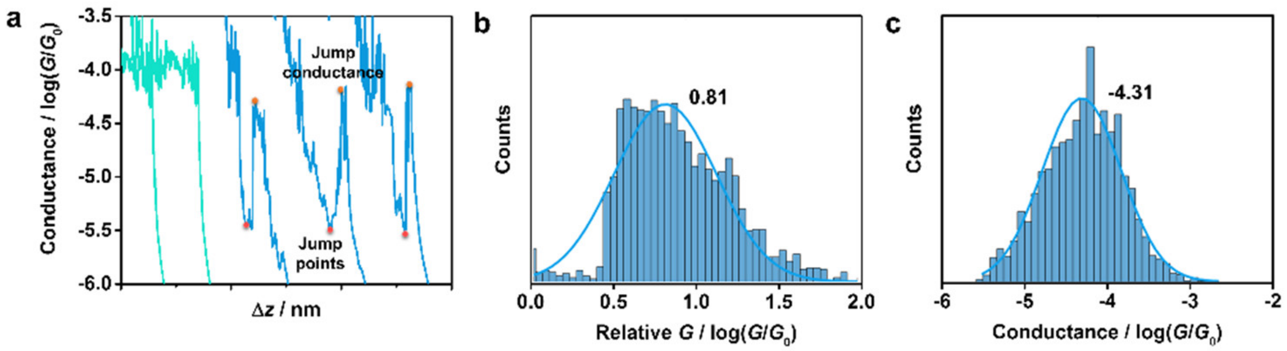

Supplementary Figure 14 | The "jump curves" experimental analysis results of $MAPbBr_3$ perovskite QDs. a, The typical individual conductance-distance traces of $MAPbBr_3$ QDs for non-jump conductance curves (green) and "jump conductance" curves (blues). The origin bright spot indicates the defined "jump conductance" and the red bright spot indicates the defined "jump points". **b,** The relative conductance (*G*) distribution histogram of the "jump curves" analyzed from Fig. 2d. **c,** The 1D conductance (*G*) distribution histogram of the "jump conductance".

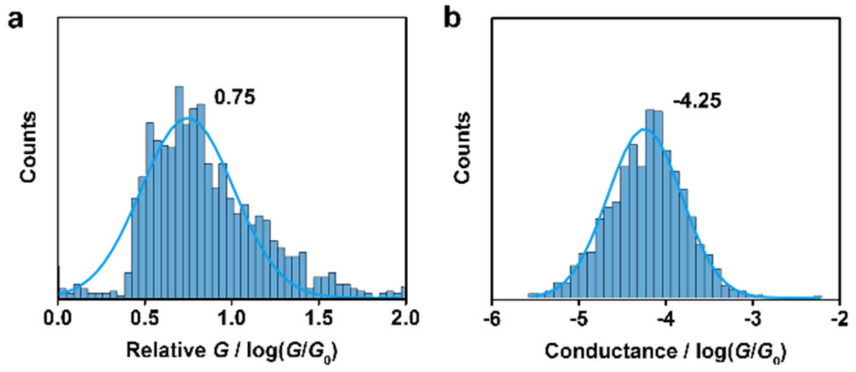

Supplementary Figure 15 | The "jump curves" experimental analysis results of $MAPbBr_{2.15}Cl_{0.85}$ perovskite QDs. a, The relative conductance (*G*) distribution histogram of the "jump curves" analyzed from Fig. 3e. **b,** The 1D conductance (*G*) distribution histogram of the "jump conductance".

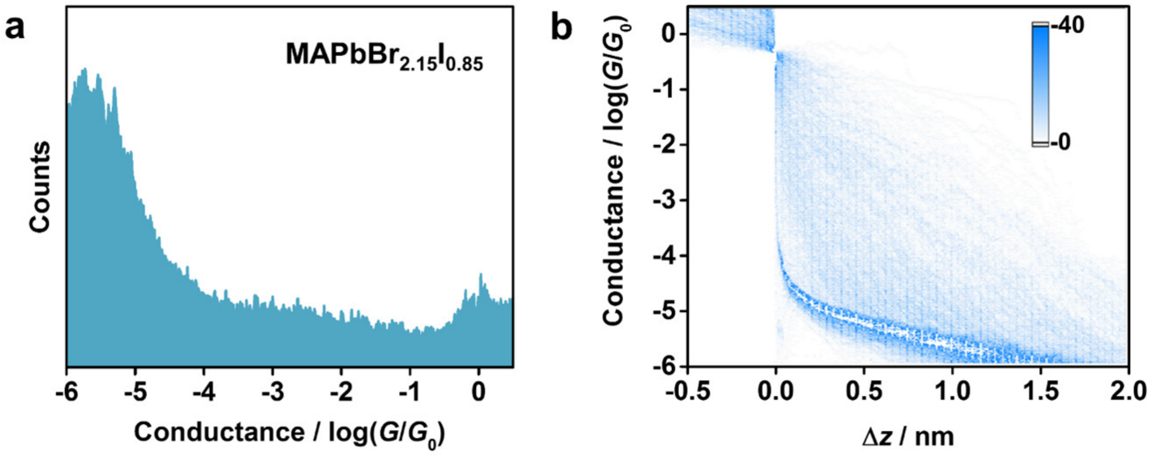

Supplementary Figure 16 | The MCBJ measurement of $MAPbBr_{2.15}I_{0.85}$ QDs. a, 1D conductance histograms for perovskite QDs $MAPbBr_{2.15}I_{0.85}$. **b,** The 2D conductance versus relative distance (Δz) histogram for perovskite QDs $MAPbBr_{2.15}I_{0.85}$.

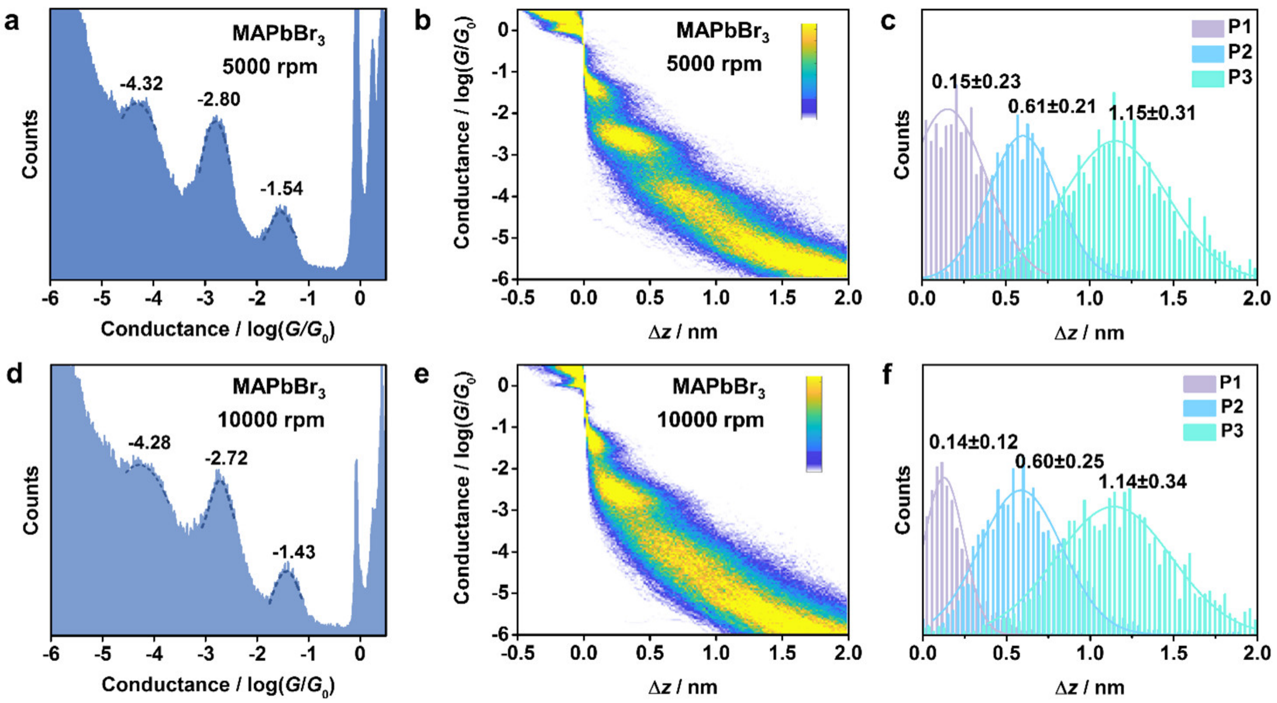

Supplementary Figure 17 | The MCBJ measurements of $MAPbBr_3$ QDs with different centrifugal speeds. a. 1D Conductance histogram constructs without data selection for $MAPbBr_3$ with the centrifugal speed of 5000 rpm. The conductance-distance traces are recorded approximately 2500 traces. **b.** All-data-points 2D conductance versus relative distance (Δz) histogram for $MAPbBr_3$ with the centrifugal speed of 5000 rpm. **c.** The displacement distributions of three plateaus for $MAPbBr_3$ with the centrifugal speed of 5000 rpm. **d.** 1D Conductance histogram constructs without data selection for $MAPbBr_3$ with the centrifugal speed of 10000 rpm. approximately 2500 conductance-distance traces are recorded. **e.** All-data-points 2D conductance versus relative distance (Δz) histogram for $MAPbBr_3$ with the centrifugal speed of 10000 rpm. **f.** The displacement distributions of three plateaus for $MAPbBr_3$ with the centrifugal speed of 10000 rpm.

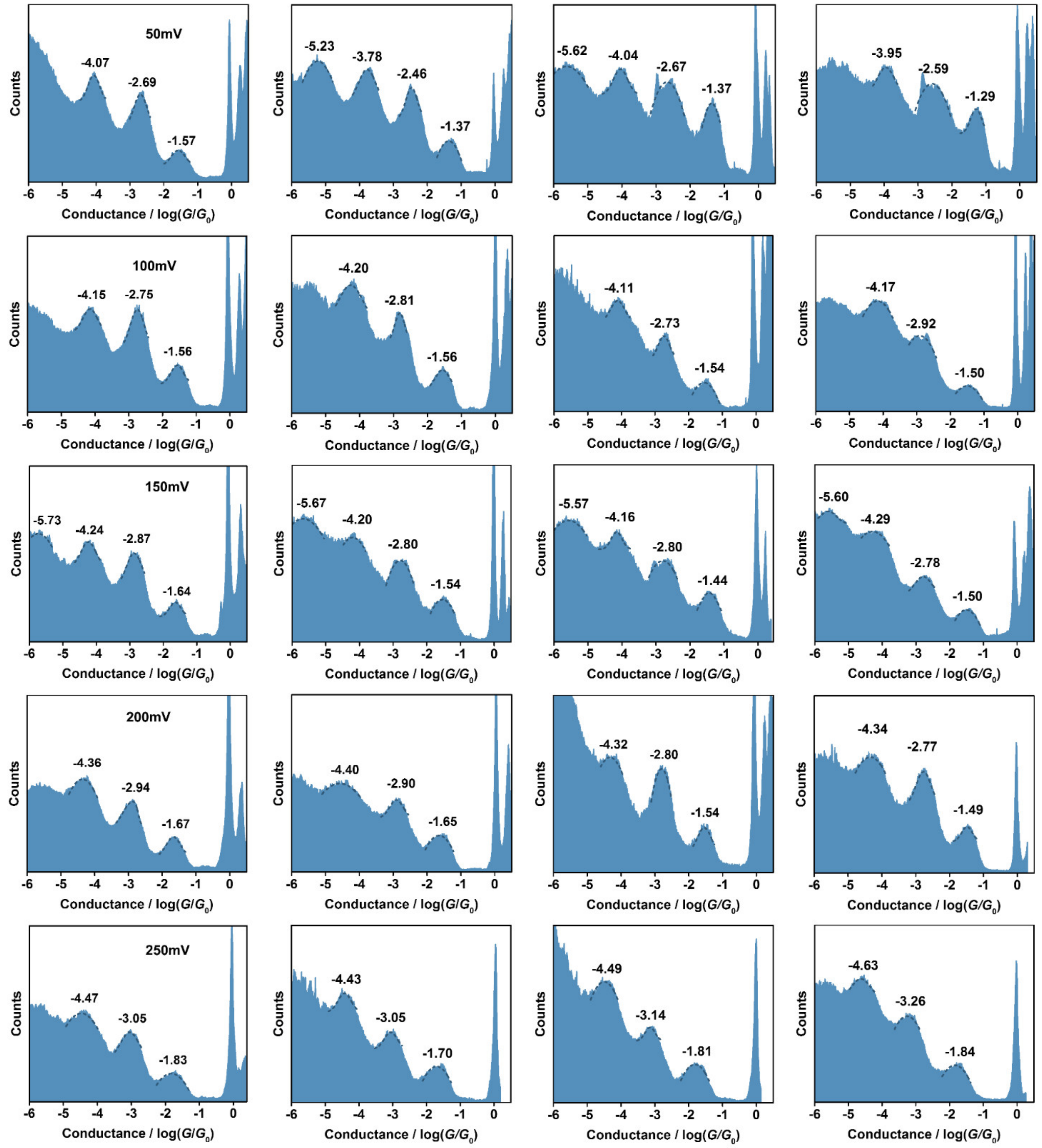

Supplementary Figure 18 | 1D conductance histograms of $MAPbBr_3$ QDs at different bias voltages.

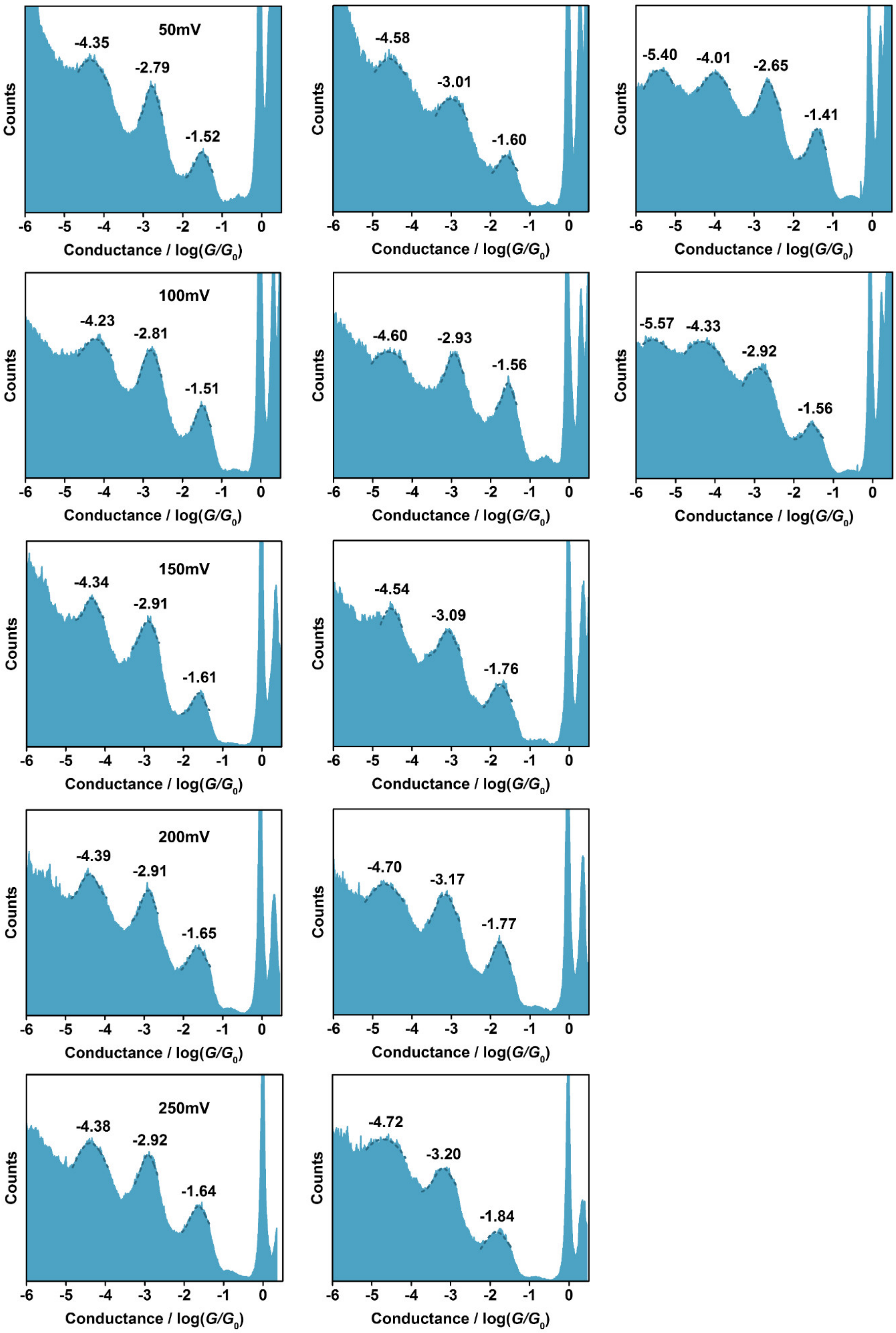

Supplementary Figure 19 | 1D conductance histograms of $MAPbBr_{2.15}Cl_{0.85}$ QDs at different bias voltages.

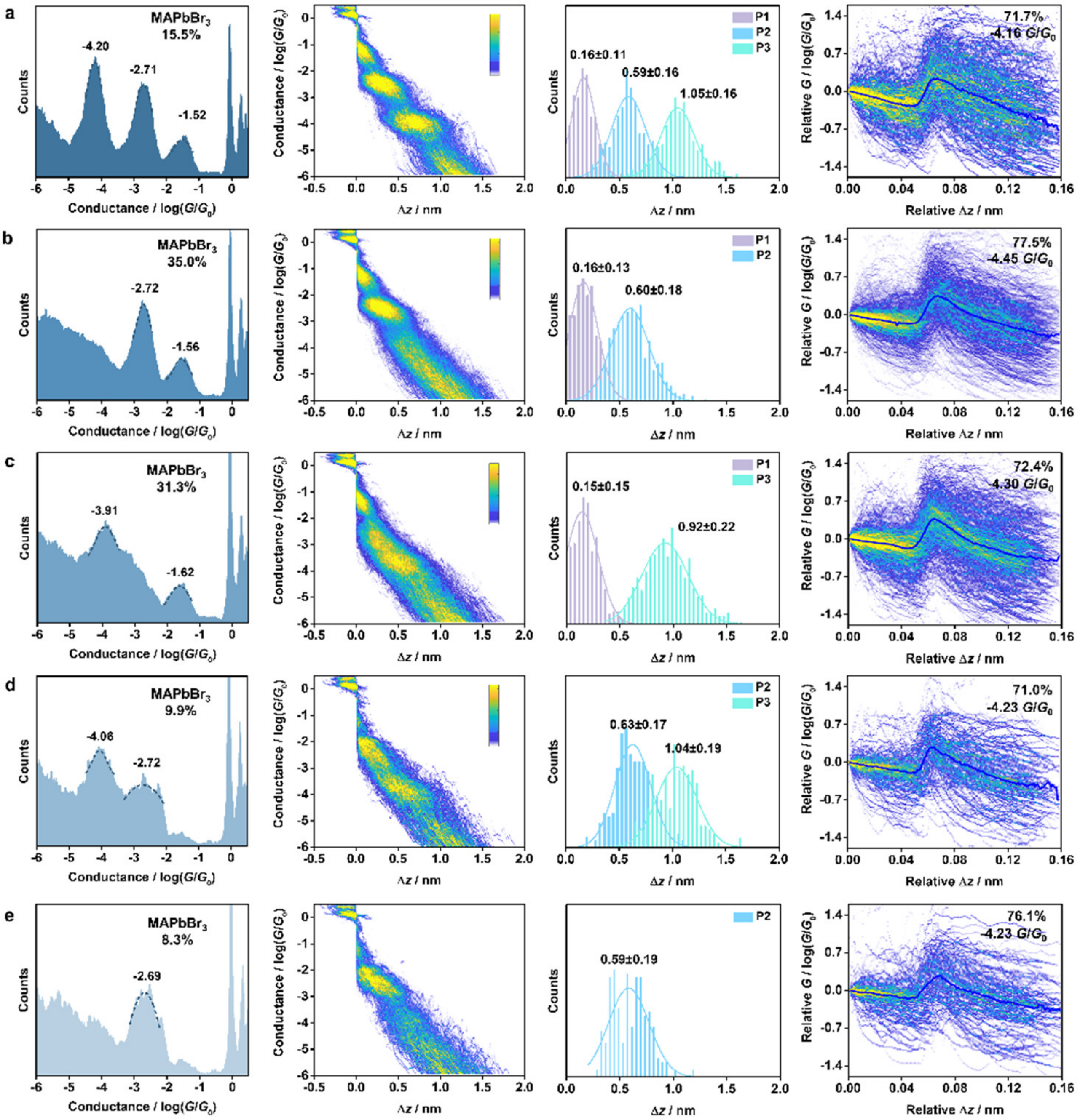

Supplementary Figure 20 | The classification results of $MAPbBr_3$ QDs using spectral clustering algorithms. a, 15.5% of traces with three successive conductance plateaus. **b,** 35.0% of traces with the highest conductance (HC) and middle conductance (MC). **c,** 31.3% of traces with the HC and the lowest conductance (LC). **d,** 9.9% of traces with MC and LC. **e,** 8.3% of traces with only MC.

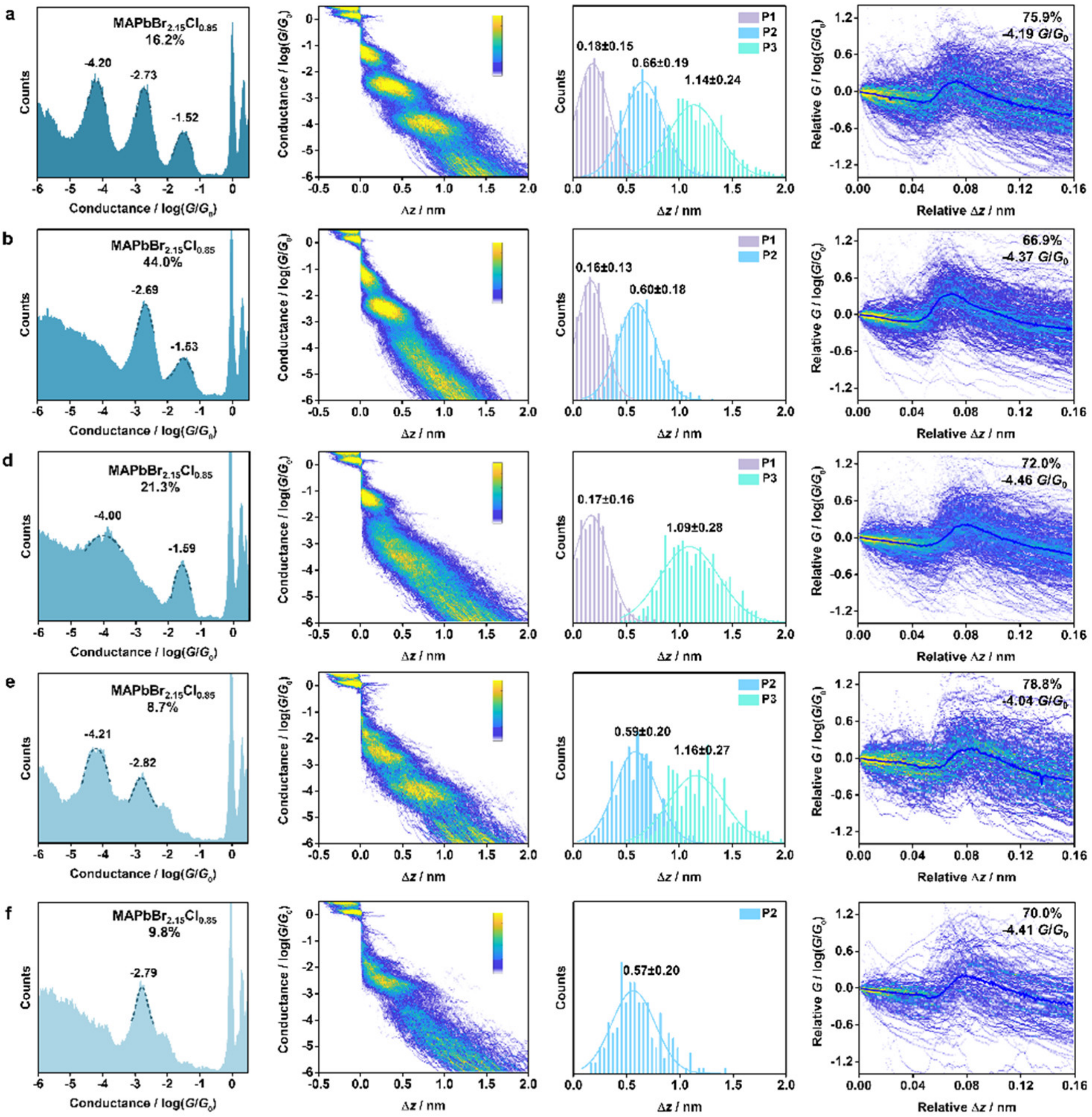

Supplementary Figure 21 | The classification results of $MAPbBr_{2.15}Cl_{0.85}$ QDs using spectral clustering algorithms. a, 16.2% of traces with three successive conductance plateaus. **b,** 44.0% of traces with the highest conductance (HC) and middle conductance (MC). **c,** 21.3% of traces with the HC and the lowest conductance (LC). **d,** 8.7% of traces with MC and LC. **e,** 9.8% of traces with only MC.

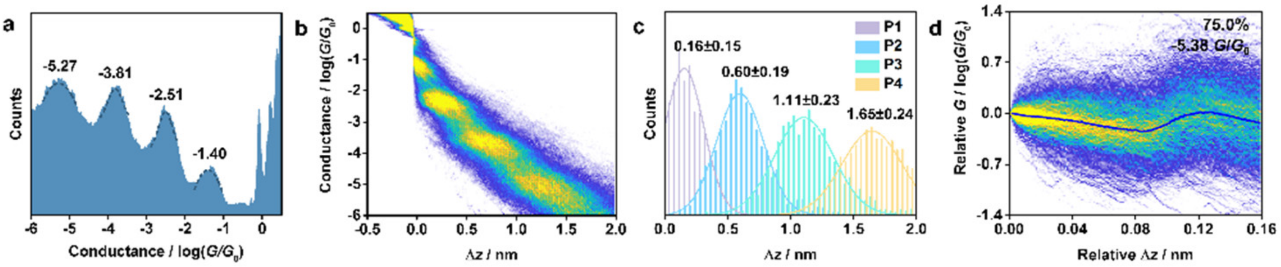

Supplementary Figure 22 | The classification results of $MAPbBr_3$ QDs with four conductance plateaus using spectral clustering algorithms. a, 1D Conductance histogram constructs without data selection for $MAPbBr_3$ QDs. **b,** All-data-points 2D conductance versus relative distance (Δz) histogram for $MAPbBr_3$ QDs. **c,** The displacement distributions of three plateaus for $MAPbBr_3$ QDs. **d,** 2D relative conductance (G) verse relative displacement (Δz) histogram of the "jump curves" for $MAPbBr_3$ QDs.

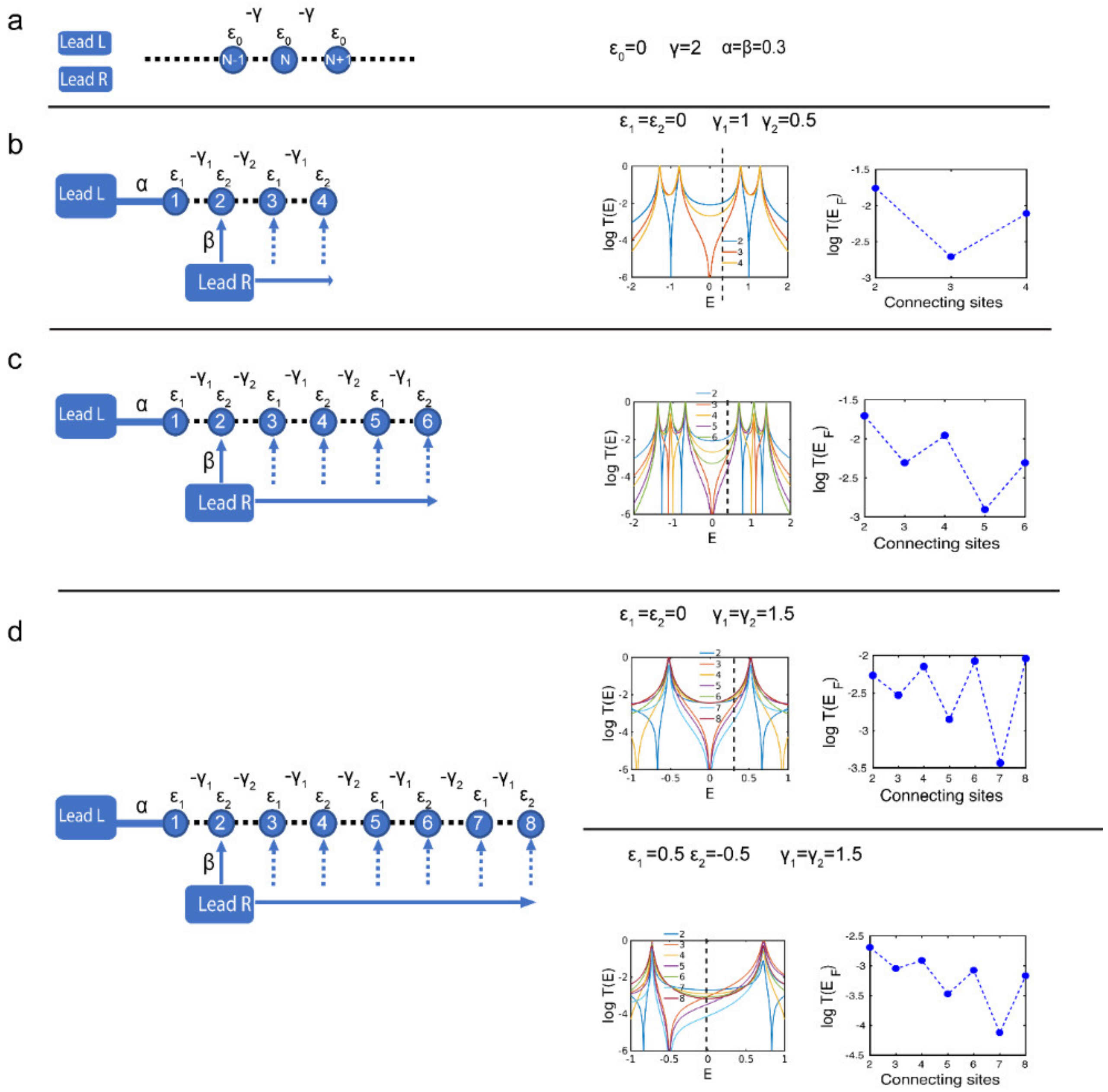

Supplementary Figure 23 | The tight-binding model. a, The schematic of the uniform left and right lead parameter used in our simulations. **b,** The schematic of model with 4 sites diatomic chain. Site 1 is connected to Lead L, sites 2-4 are connected to Lead R sequentially. On the right side, the corresponding transmission spectra and the T(E_F) are plotted. The Fermi energy chosen is indicated by the dashed line. **c,** the schematic of model with 6 sites diatomic chain. Site 1 is connected to Lead L, sites 2-6 are connected to Lead R sequentially. On the right side, the corresponding transmission spectra and the T(E_F) are plotted. The Fermi energy chosen is indicated by the dashed line. **d,** the schematic of model with 8 sites diatomic chain. Site 1 is connected to Lead L, sites 2-8 are connected to Lead R sequentially. On the right side, the corresponding transmission spectra and the T(E_F) are

plotted. The Fermi energy chosen is indicated by the dashed line. Different from the case we discussed in manuscript, where the ε is same while the γ is different, two cases are discussed here, 1- both the ε and γ are same. 2- the γ is same while the ε is different.

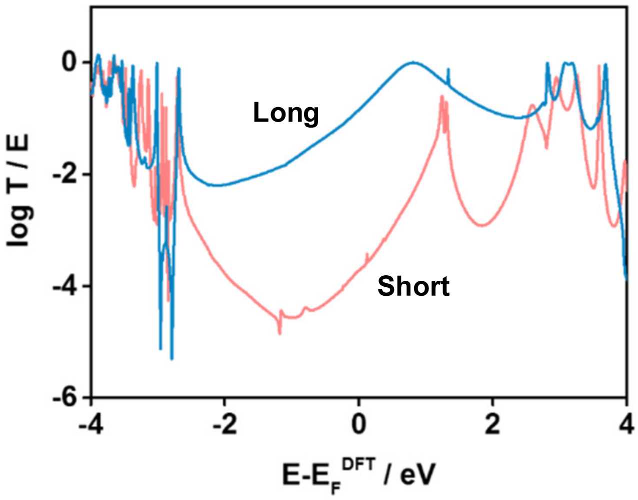

Supplementary Figure 24 | Transmission spectra for the two connections in Fig. 1b, showing that the longer path is more conductive than the shorter one.

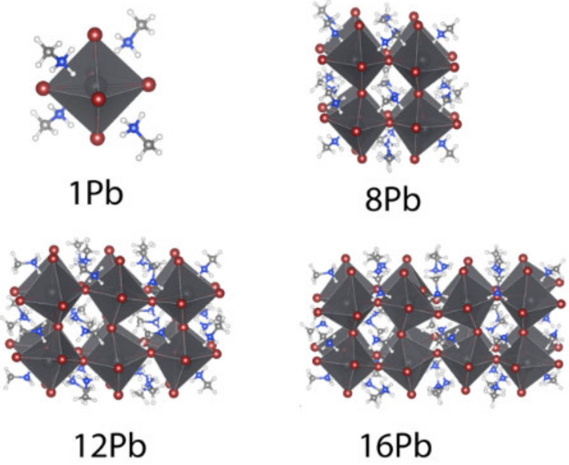

Supplementary Figure 25 | The relaxed conformations for neutral 1Pb, 8Pb, 12Pb and 16Pb $MAPbBr_3$ clusters. For 1Pb (MA_4PbBr_6), 8Pb ($MA_{20}Pb_8Br_{36}$) and 12Pb ($MA_{28}Pb_{12}Br_{52}$), we use the lowest energy coordinates in the literature,[33] and then relax these structures using SIESTA. We construct 16Pb ($MA_{36}Pb_{16}Br_{68}$) by adding another four octahedron units based on the 12Pb, and then relax.

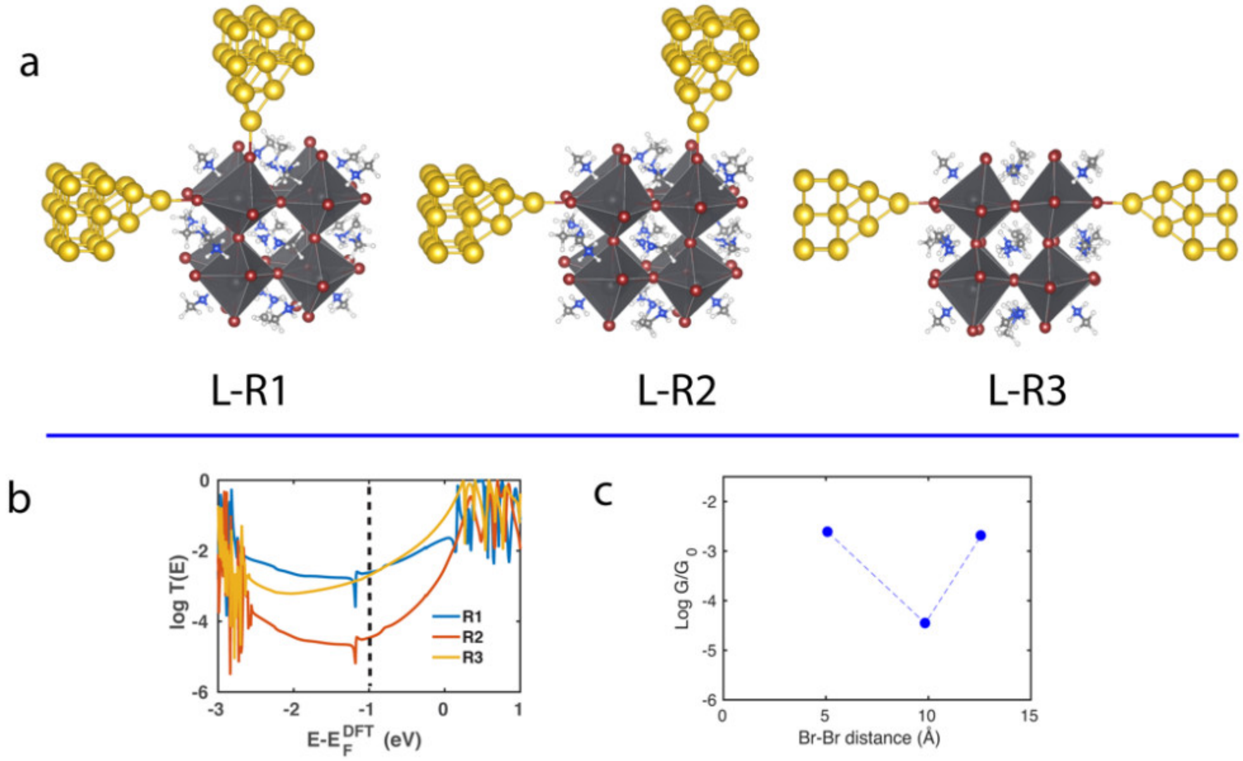

Supplementary Figure 26 | The DFT calculation of 8Pb $MAPbBr_3$ cluster. a, The conformations for 8Pb $MAPbBr_3$ cluster embedded in two gold electrodes with 3 different connectivity L-R1, L-R2 and L-R3. **b,** The corresponding transmission spectra. **c,** The room temperature conductance with the E_F=-1 eV as indicated by the black dashed line in b.

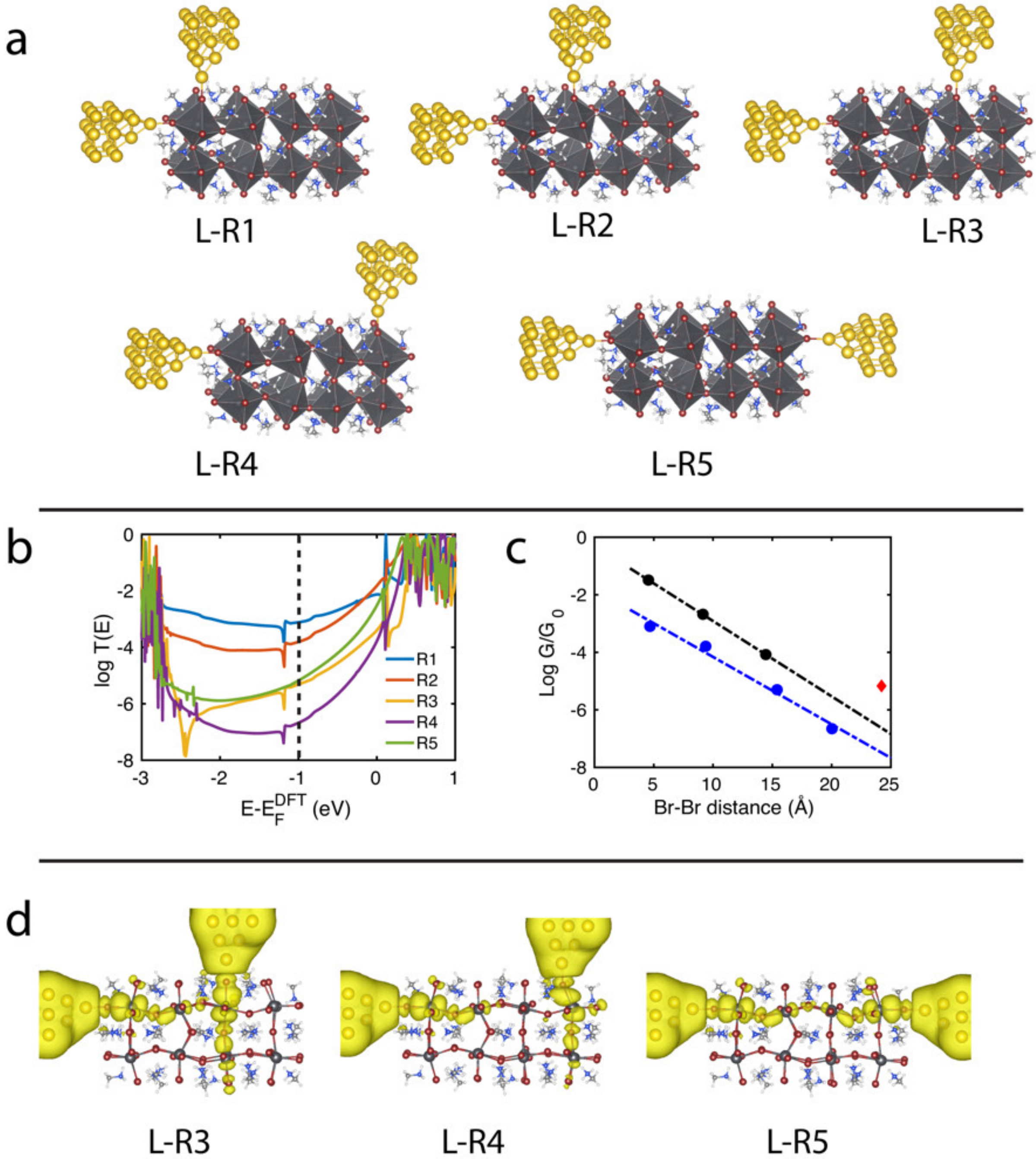

Supplementary Figure 27 | The DFT calculation of 16Pb $MAPbBr_3$ cluster. a, The conformations for 16Pb $MAPbBr_3$ cluster embedded in junctions with 5 different connectivity L-R1, L-R2, L-R3, L-R4 and L-R5. **b,** The corresponding transmission spectra. **c,** The room temperature conductance versus their distances with the E_F = -1 eV as indicated by the black dashed line in b, the experimental results are also put here for comparison. The jump conformation L-R5 is indicated by the red diamond. **d,** The LDOS with yellow color in the energy window from –1.5 eV to -0.5 eV for 'L-R3' 'L-R4' and 'L-R5' separately at the isosurface 0.00004.

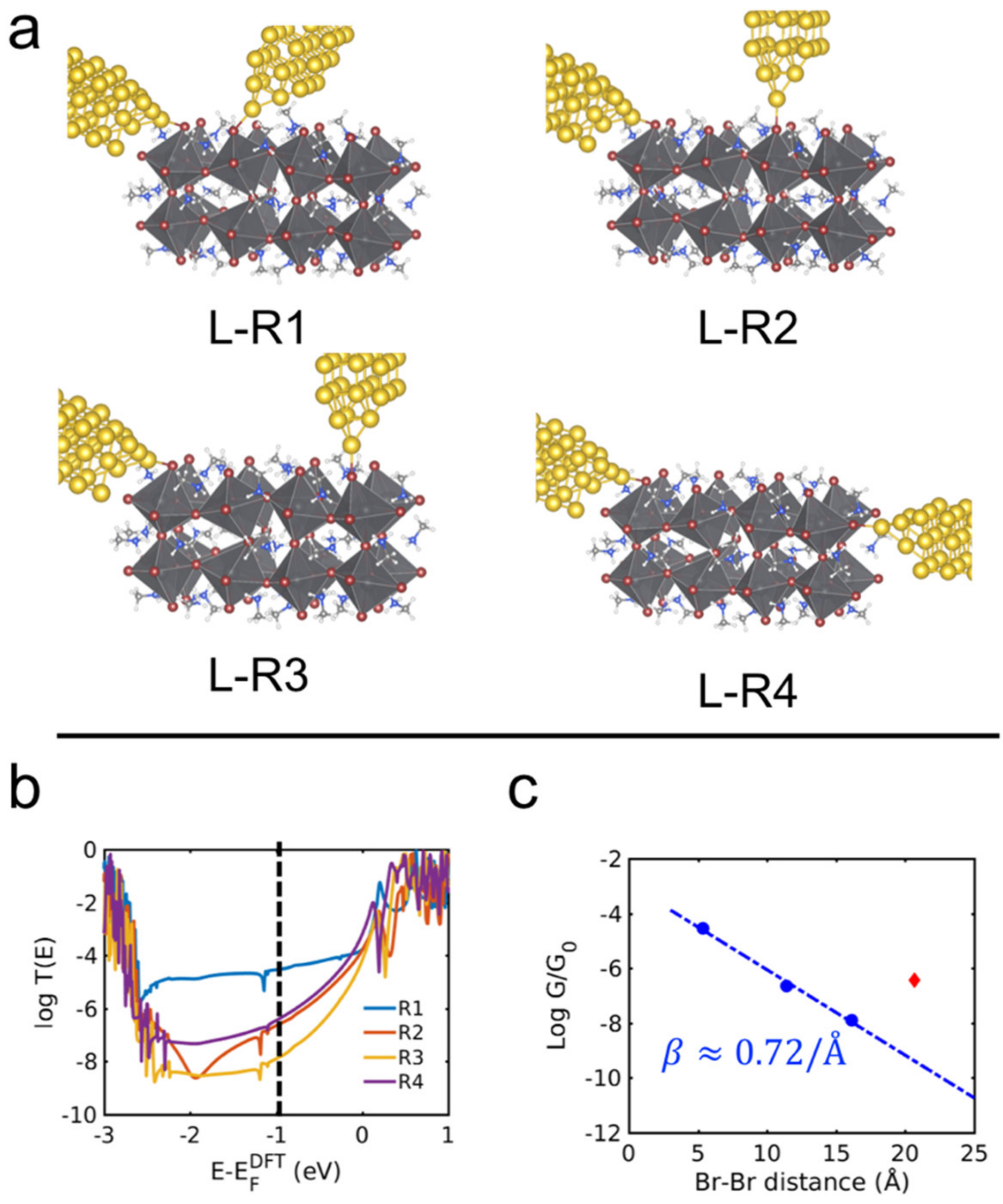

Supplementary Figure 28 | The DFT calculation of pulling process for 16Pb $MAPbBr_3$ cluster with a cross-plane manner. a, The conformations for 16Pb $MAPbBr_3$ cluster embedded in junctions with 4 different connectivity L-R1, L-R2, L-R3, L-R4. **b,** The corresponding transmission spectra. **c,** The room temperature conductance versus their distances with the E_F = -1 eV as indicated by the black dashed line in b. The jump conformation L-R4 is indicated by the red diamond.

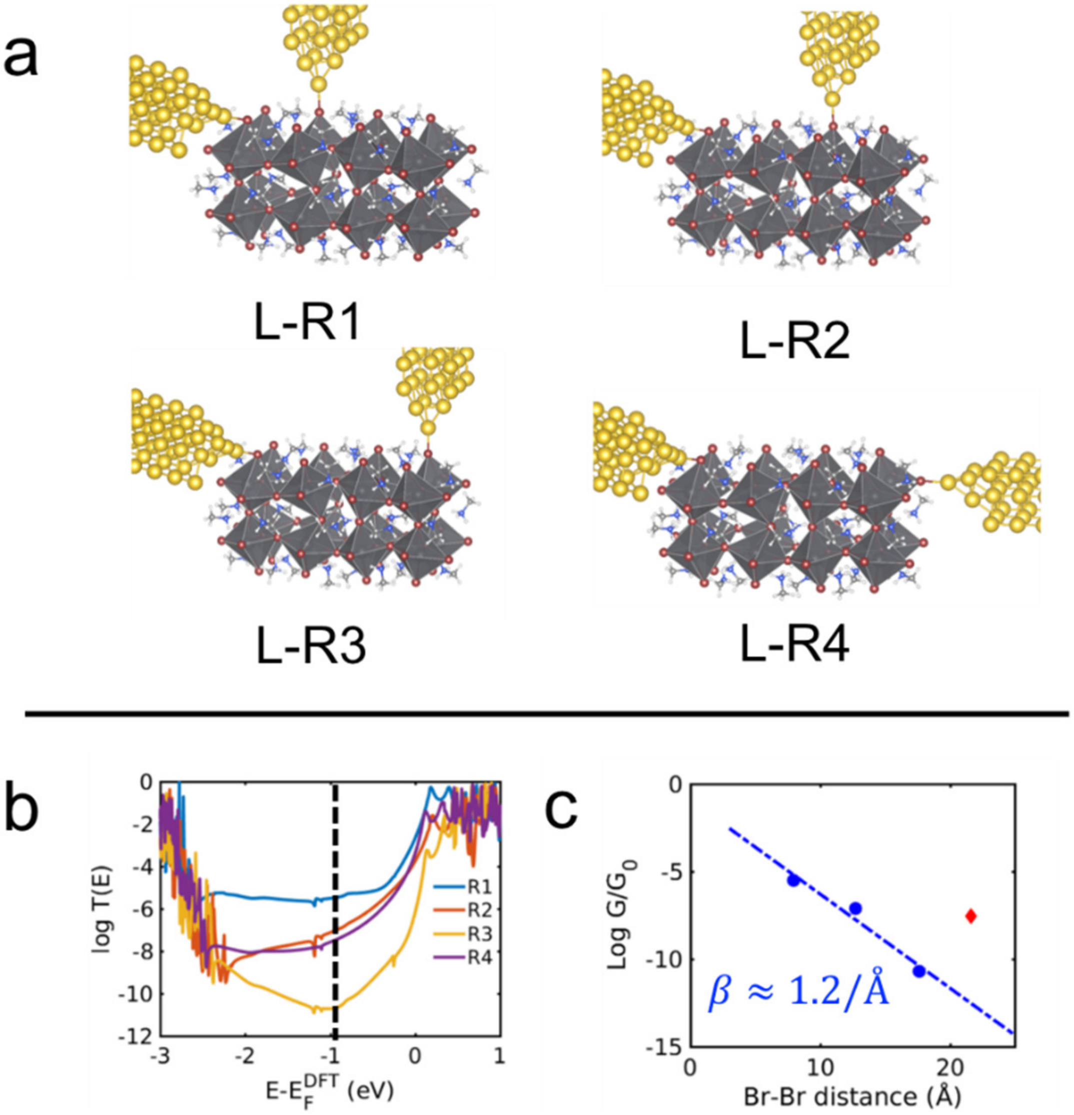

Supplementary Figure 29 | The DFT calculation of nonlinear pulling process for 16Pb $MAPbBr_3$ cluster with a cross-plane manner. a, The conformations for 16Pb $MAPbBr_3$ cluster embedded in junctions with 3 different connectivity L-R1, L-R2, L-R3 and L-R4. **b,** The corresponding transmission spectra. **c,** The room temperature conductance versus their distances with the E_F = -1 eV as indicated by the black dashed line in b. A larger β factor is obtained due to the higher energy barrier and the jump conformation L-R4 is indicated by the red diamond.

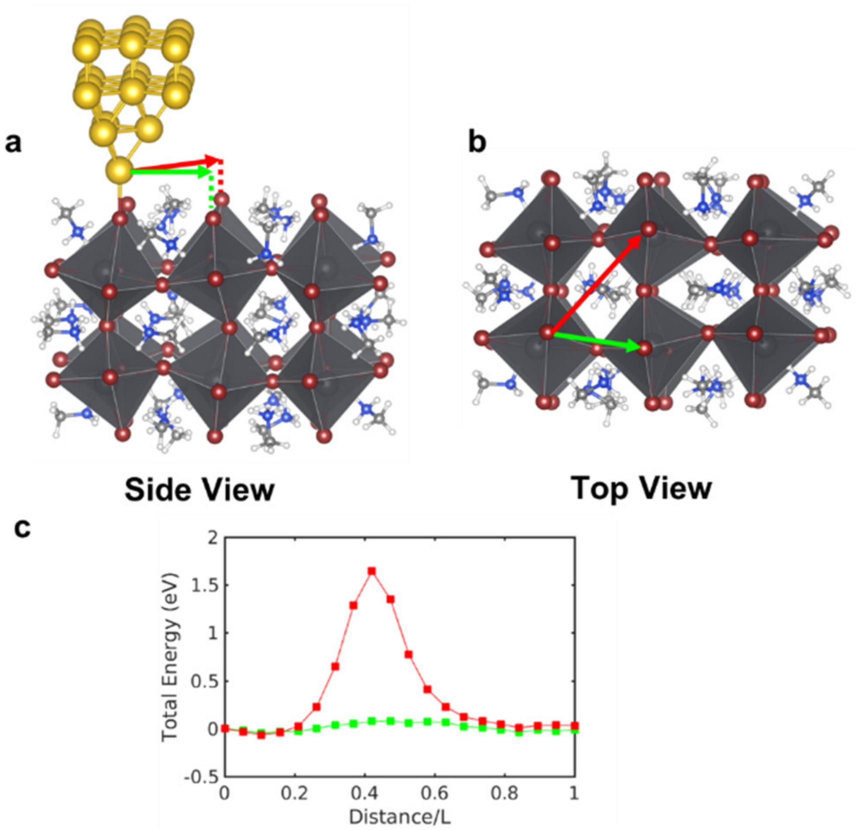

Supplementary Figure 30 | Energy landscapes versus the displacement of gold electrode along two different directions. a, Side view of 2x2x3 12Pb $MAPbBr_3$ with one gold electrode. Two sliding directions are indicated by green and red arrows separately. **b,** Top view. The gold electrode is not displayed for clarity**. c,** Profiles of the total energies when moving the gold lead along these two directions with a fixed height (2.76 Å) from the line formed by the two Br atoms. L is the distance between two Br atoms along the sliding direction.

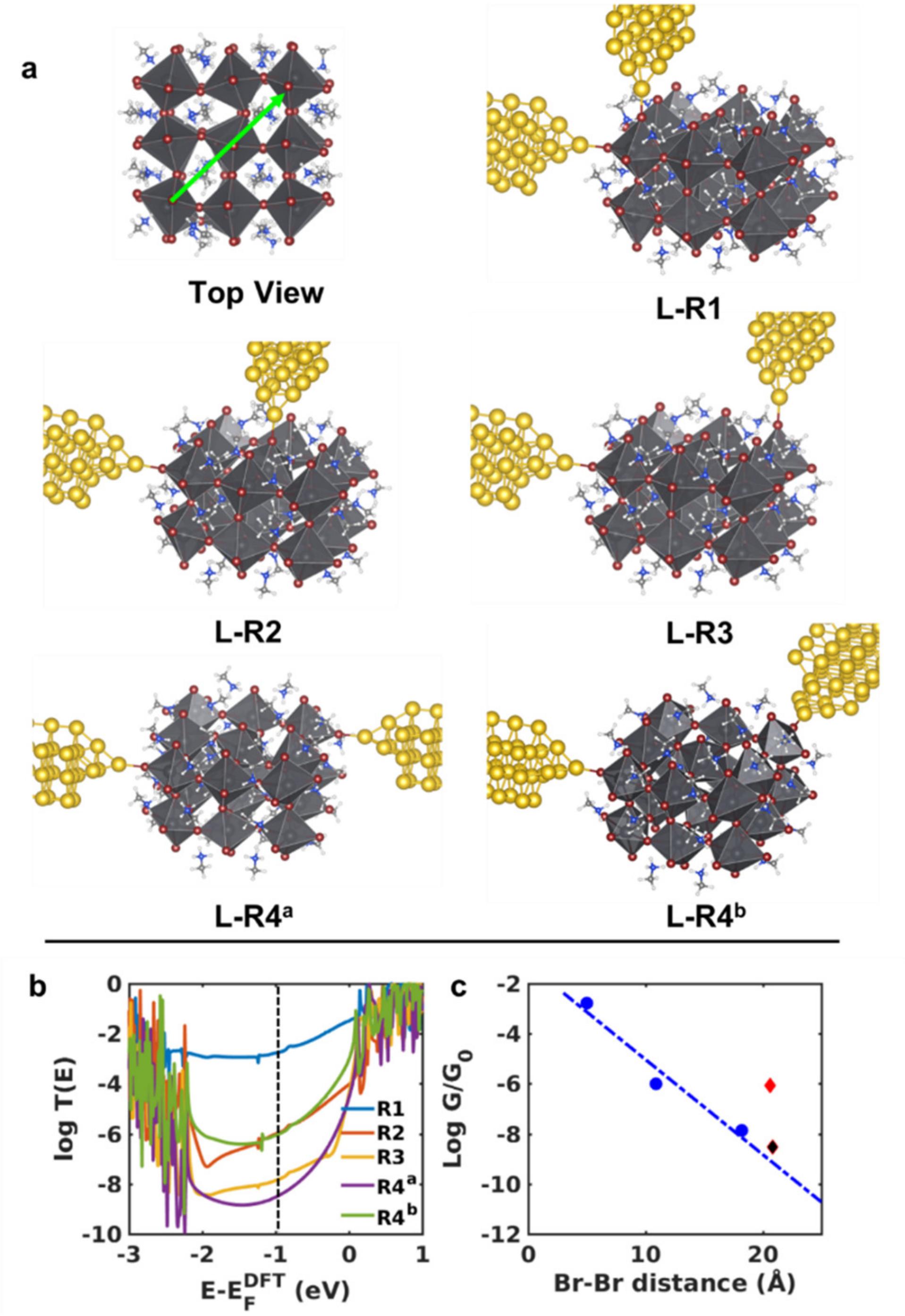

Supplementary Figure 31 | The DFT calculation of 18Pb $MAPbBr_3$ cluster when sliding along the diagonal direction. a, The top view of 18Pb cluster, the diagonal sliding direction is indicated by the green arrow. The conformations for 18Pb $MAPbBr_3$ cluster embedded in two gold electrodes with 5 different connectivity L-R1, L-R2, L-R3, L-R4^a and L-R4^b. **b-c,** The corresponding transmission spectra and the room temperature conductance with the E_F = -1 eV as indicated by the black dashed line in **b**, the $\beta \approx -0.87$

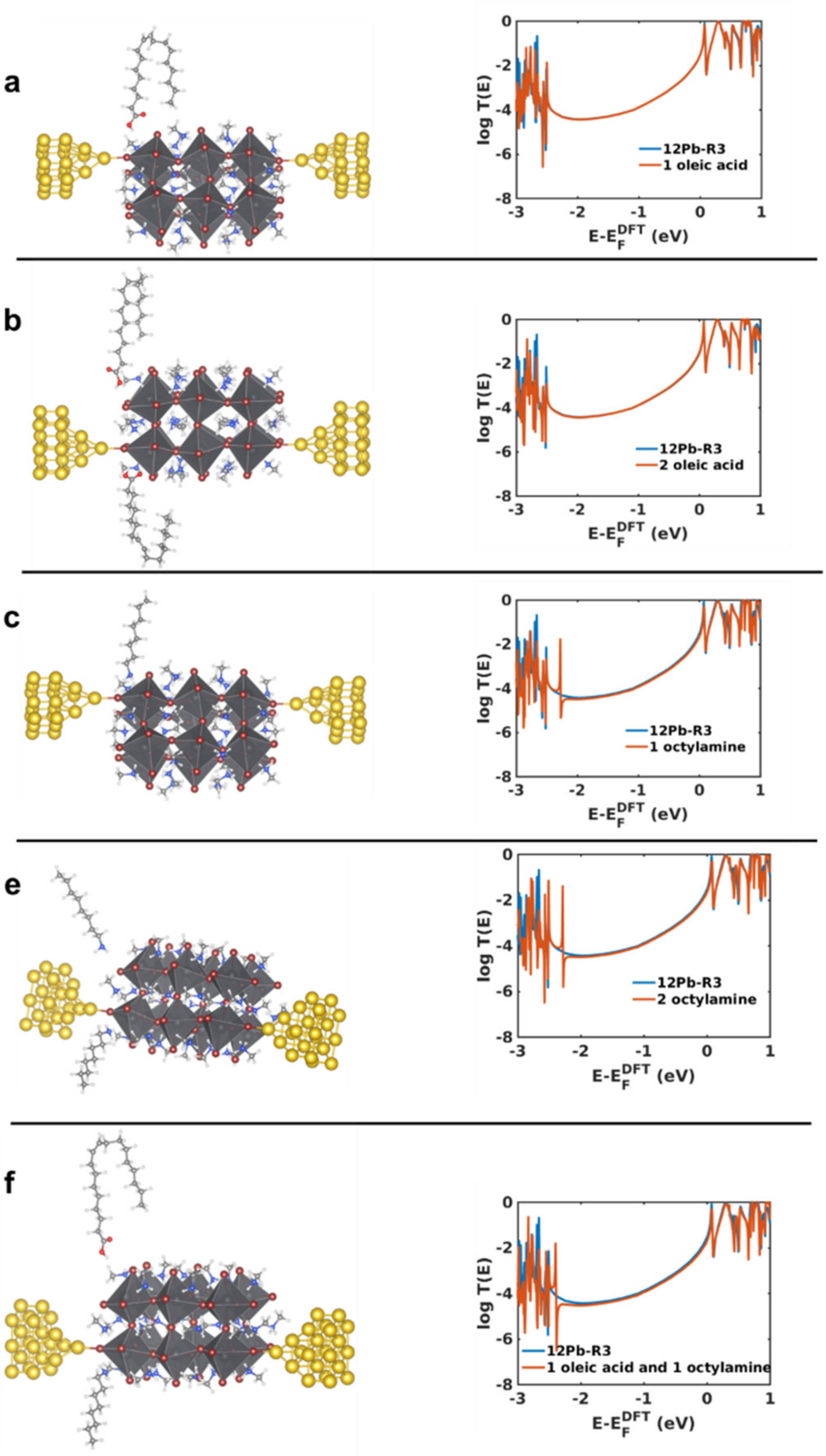

Supplementary Figure 32 | The influence of ligands on transmission functions. The conformations with ligands and the corresponding transmission functions are presented on the left side and right side respectively. For comparison, the transmission functions without ligand are also shown on the right side in blue color. **a,** 1 oleic acid **b,** 2 oleic acid **c,** 1 octylamine **d,** 2 octylamine **e,** 1 oleic acid and 1 octylamine.

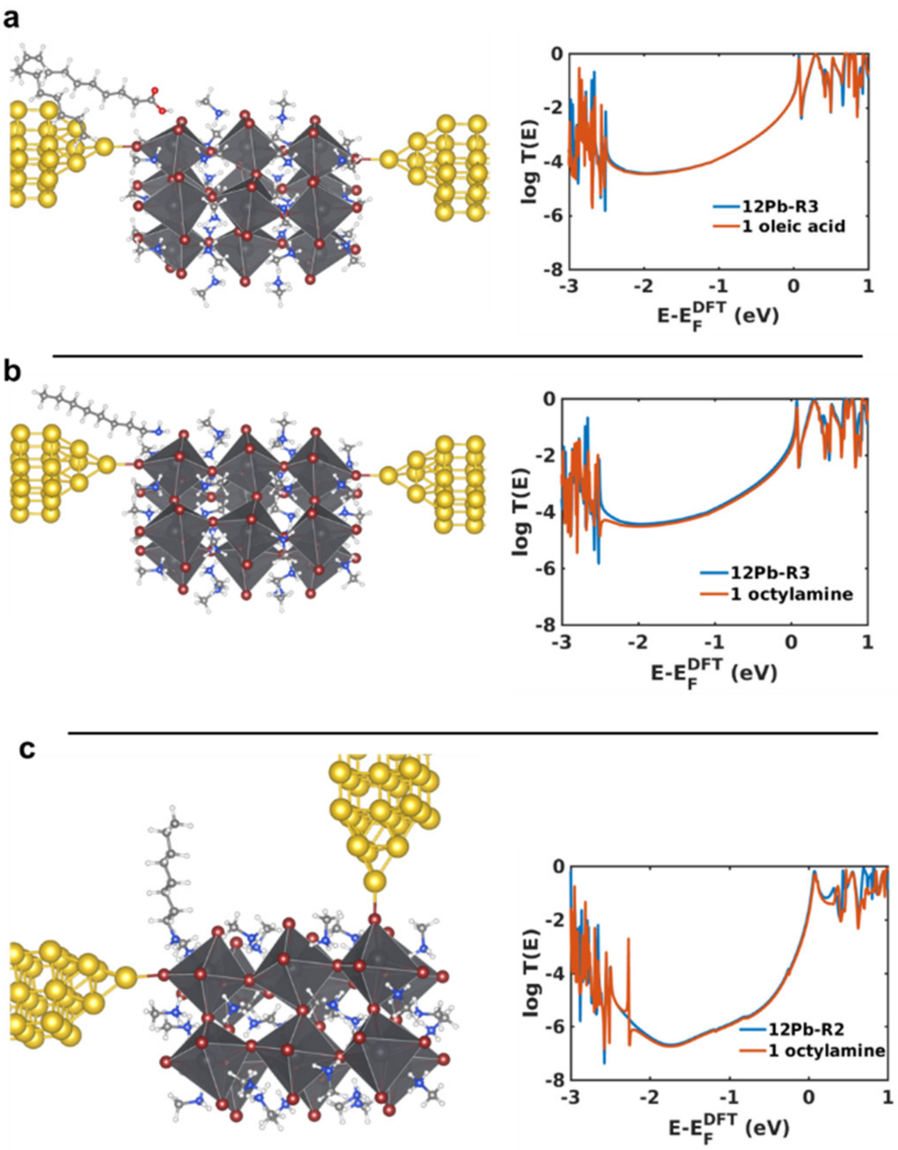

Supplementary Figure 33 | The influence of ligands on transmission functions with bridge conformations and another connectivity. The situation of the organic ligands bridging gold lead and the cluster is investigated. The conformations and the corresponding transmission functions are presented on the left side and right side respectively. For comparison, the transmission functions without ligands are also shown on the right side in blue color. **a,** 1 oleic acid **b,** 1 octylamine **c,** 1 octylamine for another connectivity."

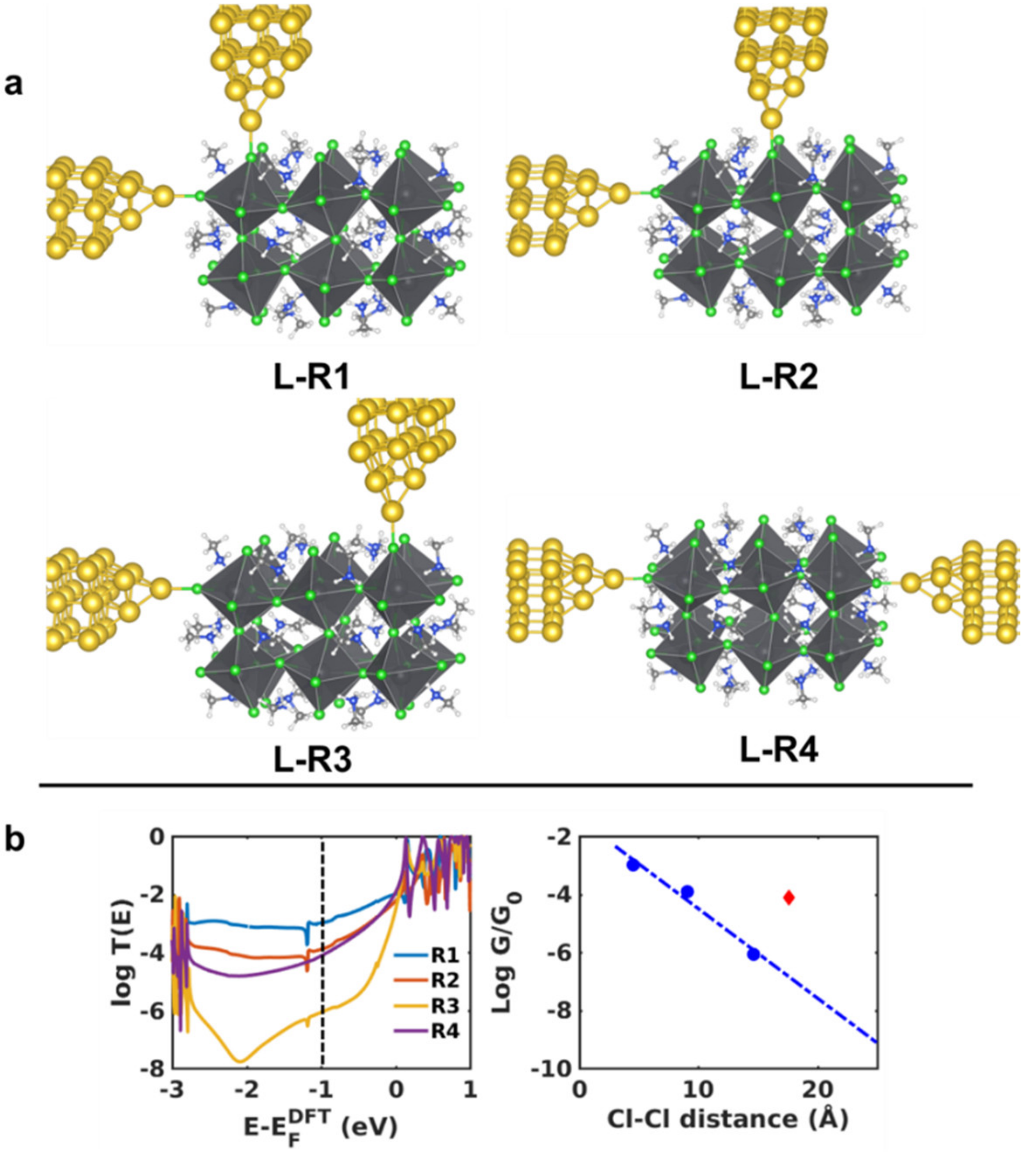

Supplementary Figure 34 | DFT calculations of 12Pb $MAPbCl_3$ cluster. a, Conformations for 12Pb $MAPbCl_3$ cluster embedded in two gold electrodes with 4 different connectivity L-R1, L-R2, L-R3 and L-R4. **b,** The corresponding transmission functions and room temperature conductances with the E_F = -1 eV relative to that estimated by DFT which is indicated by the black dashed line in the left panel.

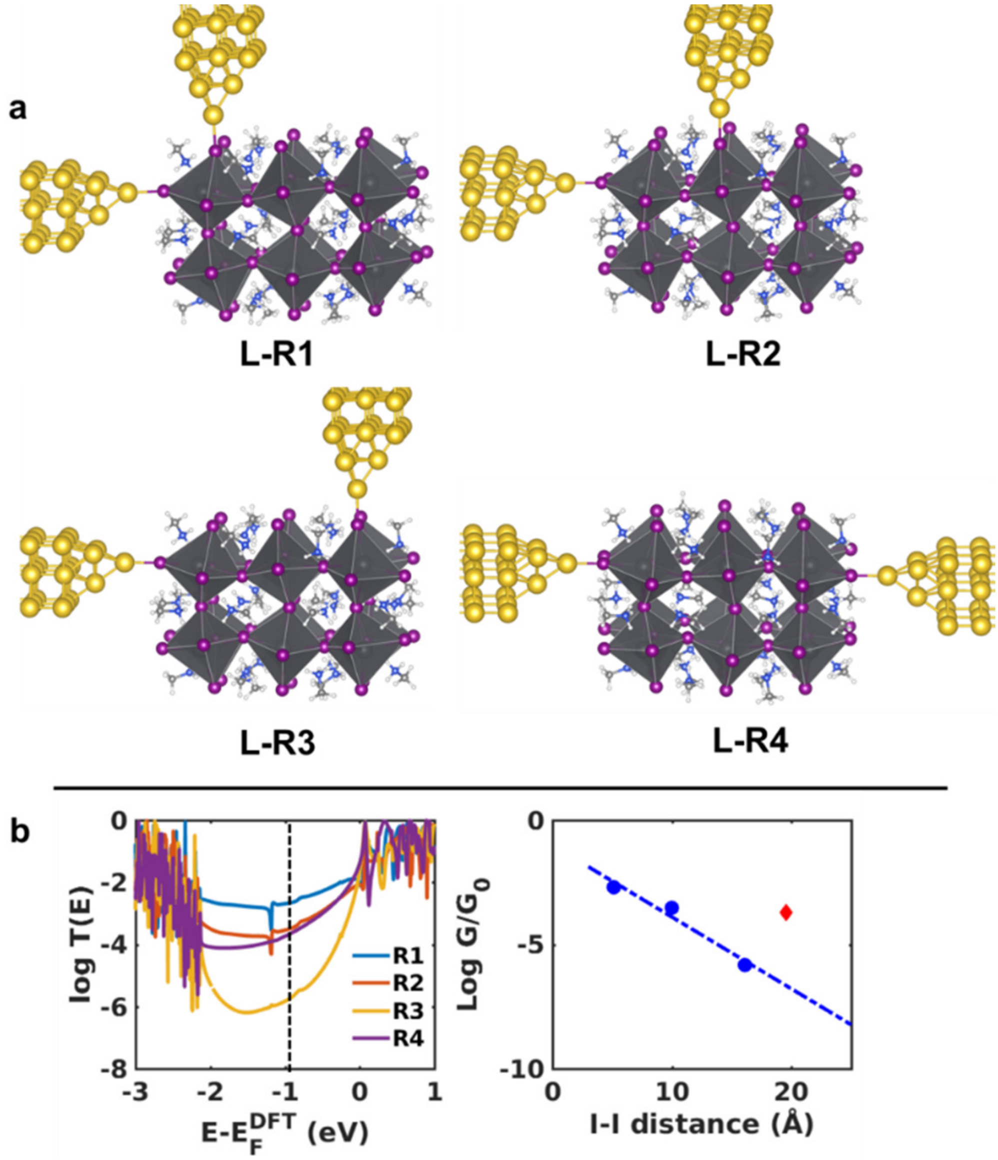

Supplementary Figure 35 | DFT calculations of 12Pb $MAPbI_3$ cluster. a, Conformations for 12Pb $MAPbI_3$ cluster embedded in two gold electrodes with 4 different connectivity L-R1, L-R2, L-R3 and L-R4. **b,** The corresponding transmission functions and room temperature conductances with the E_F = -1 eV relative to that estimated by DFT which is indicated by the black dashed line in the left panel.

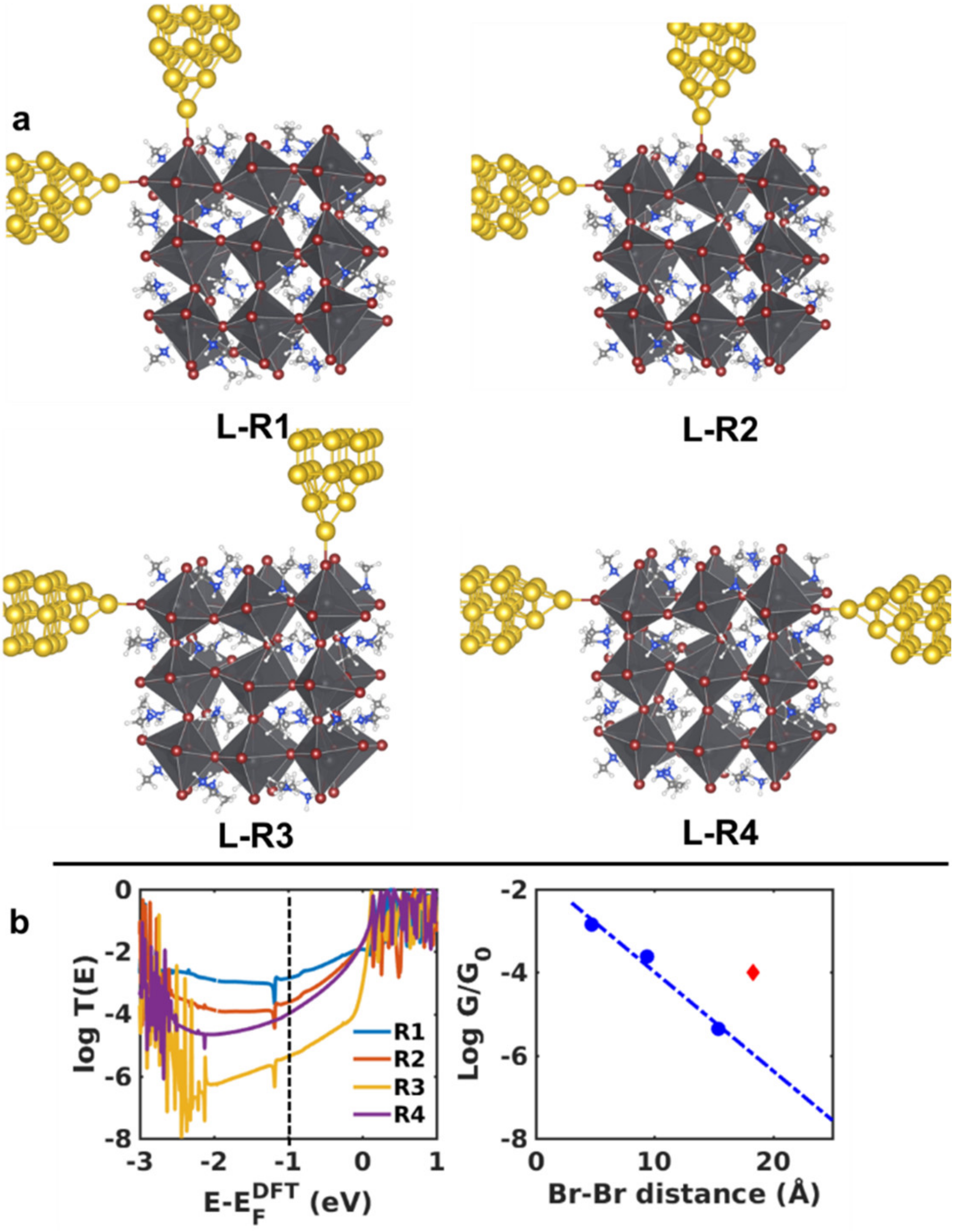

Supplementary Figure 36 | DFT calculations of 3x3x2 18Pb $MAPbBr_3$ cluster. a, Conformations for 18Pb $MAPbBr_3$ cluster embedded in two gold electrodes with 4 different connectivity L-R1, L-R2, L-R3 and L-R4. **b,** The corresponding transmission functions and room temperature conductances with the E_F = -1 eV relative to that estimated by DFT which is indicated by the black dashed line in the left panel.

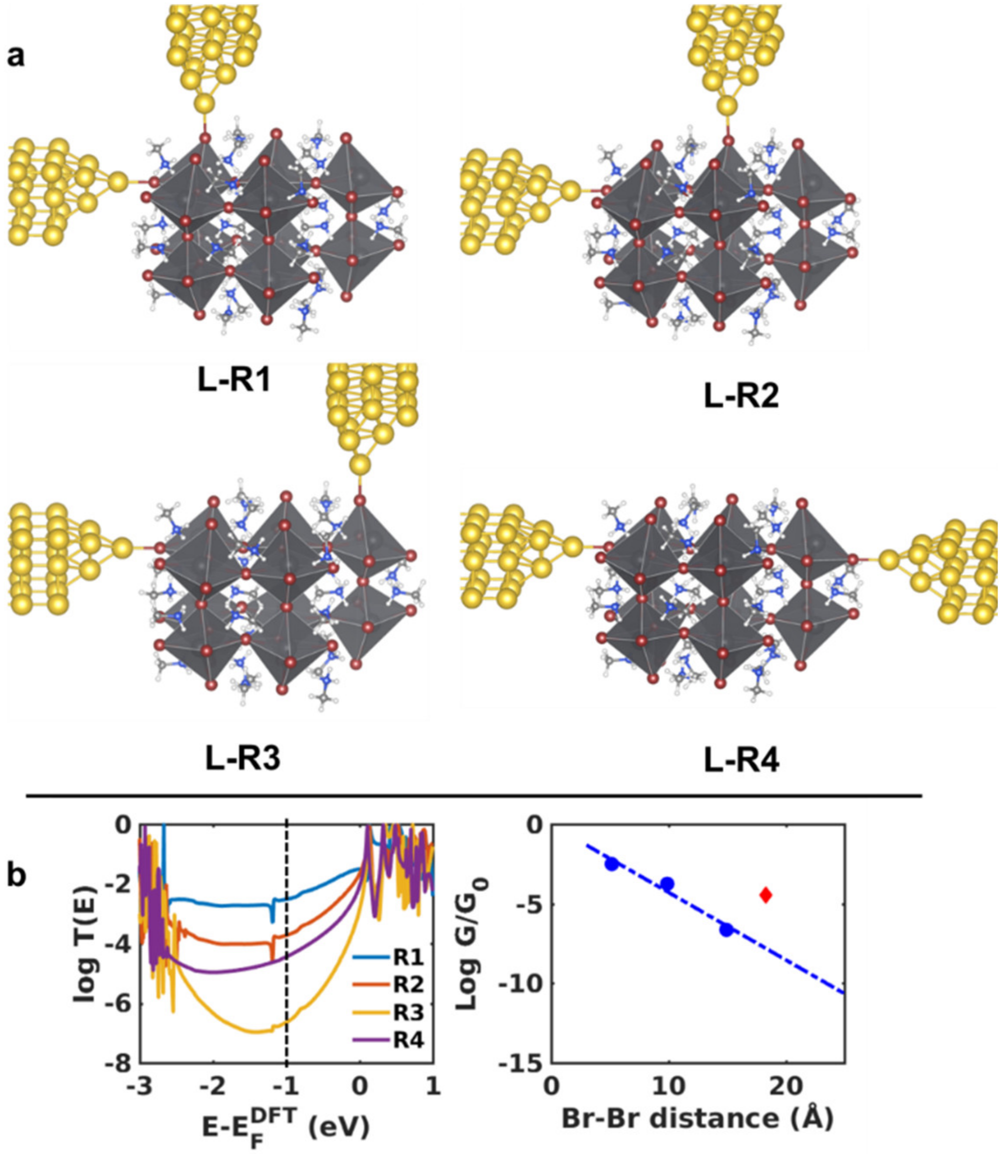

Supplementary Figure 37 | DFT calculations of 2x2x3 10Pb $MAPbBr_3$ cluster. a, Conformations for 10Pb $MAPbBr_3$ cluster embedded in two gold electrodes with 4 different connectivity L-R1, L-R2, L-R3 and L-R4. **b,** The corresponding transmission functions and room temperature conductances with the E_F = -1 eV relative to that estimated by DFT which is indicated by the black dashed line in the left panel.

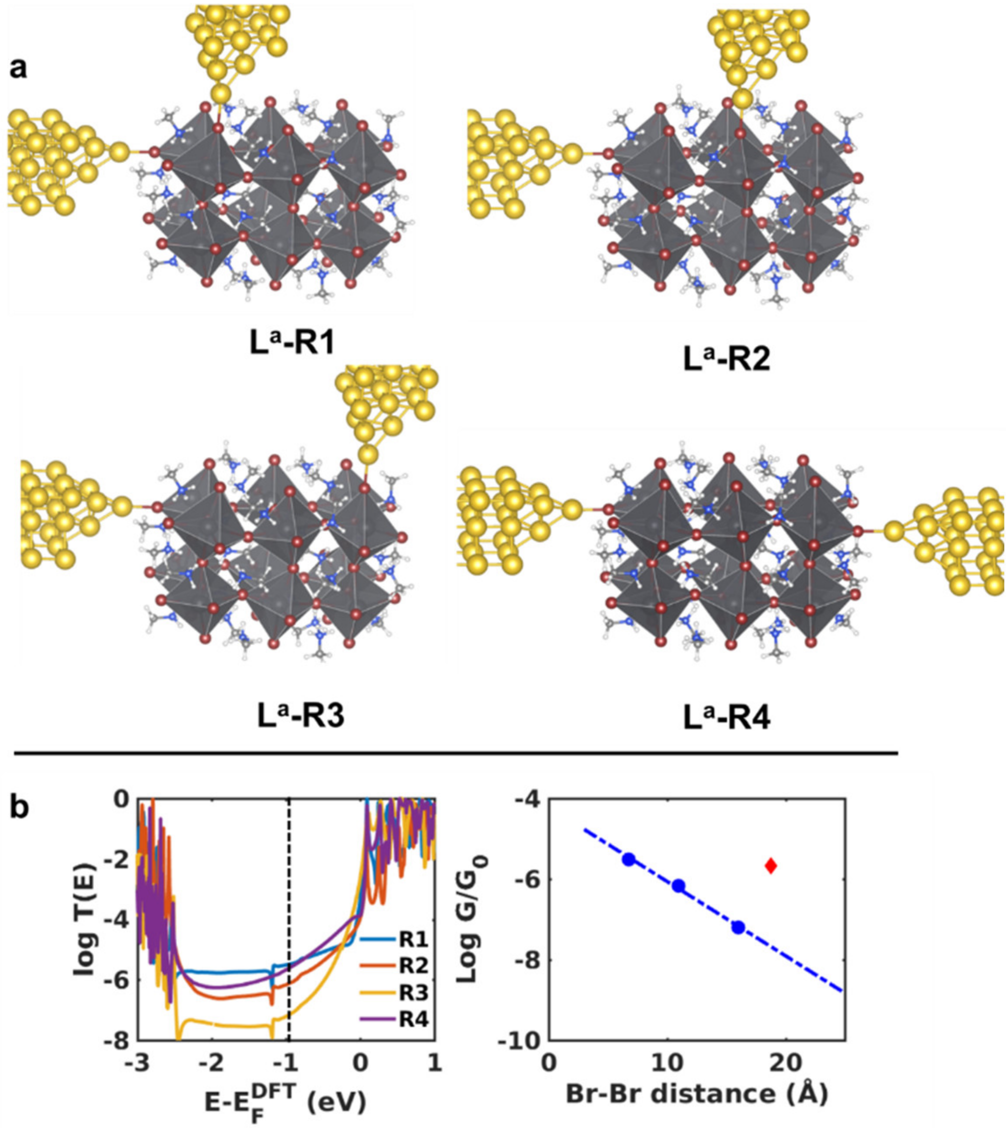

Supplementary Figure 38 | DFT calculations of 2x2x3 12Pb MAPbBr$_3$ cluster with a new left contact Br atom 'L^a'. a, Conformations for 12Pb MAPbBr$_3$ cluster embedded in two gold electrodes with 4 different connectivity L^a-R1, L^a-R2, L^a-R3 and L^a-R4. **b,** The corresponding transmission functions and room temperature conductances with the E_F = -1 eV relative to that estimated by DFT which is indicated by the black dashed line in the left panel.

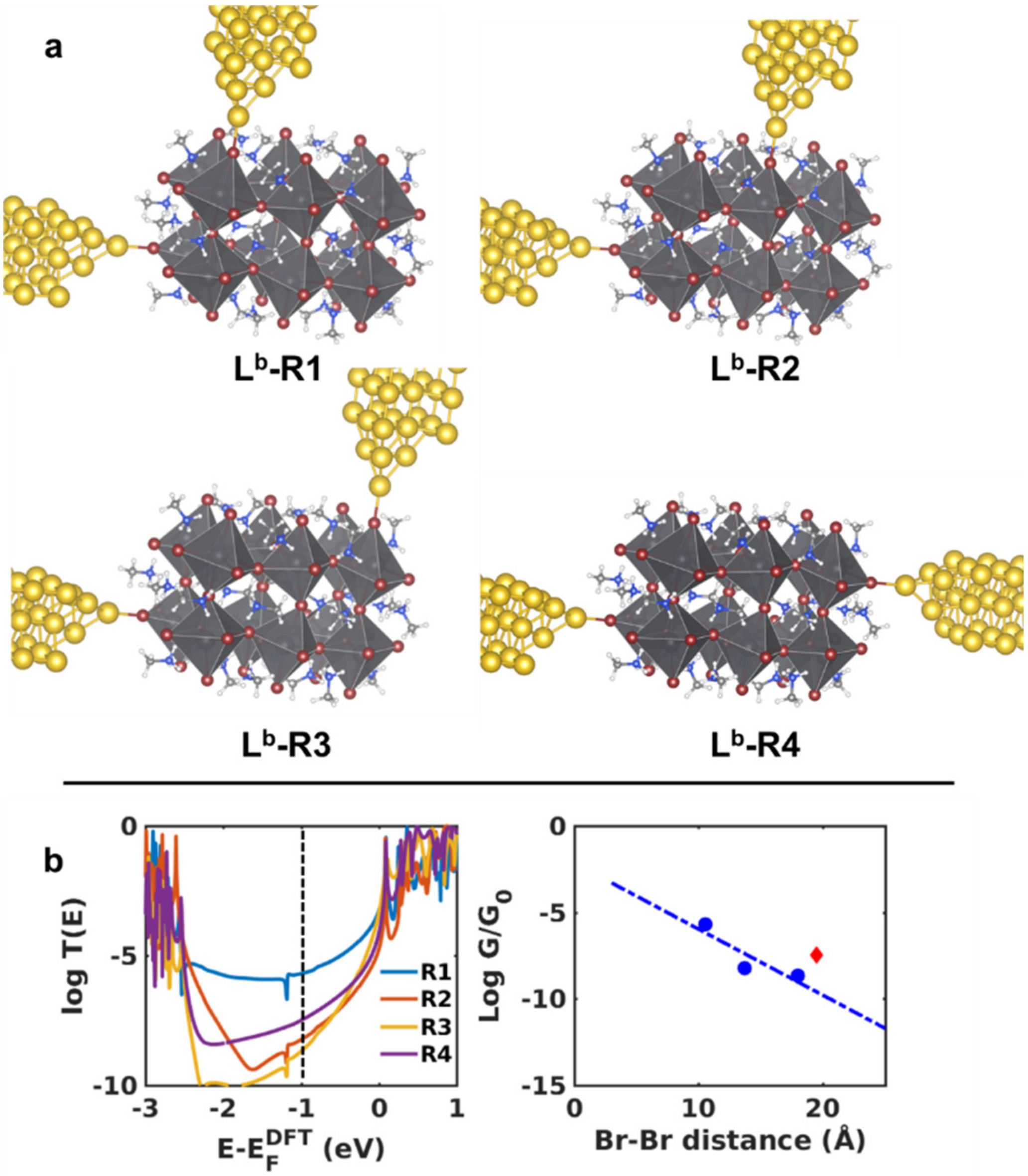

Supplementary Figure 39 | DFT calculations of 2x2x3 12Pb $MAPbBr_3$ cluster with the other new left contact Br atom 'L^b'. a, Conformations for 12Pb $MAPbBr_3$ cluster embedded in two gold electrodes with 4 different connectivity L^b-R1, L^b -R2, L^b -R3 and L^b -R4. **b,** The corresponding transmission functions and room temperature conductances with the E_F = -1 eV relative to that estimated by DFT which is indicated by the black dashed line in the left panel.

Supplementary Table 1 | The summary of experimental results for $MAPbBr_3$ QDs

Conductance / $\log(G/G_0)$ Bias voltage / mV	P1	P2	P3	P4
**50mV**	-1.57	-2.69	-4.07	
	-1.37	-2.46	-3.78	-5.23
	-1.37	-2.67	-4.04	-5.62
	-1.49	-2.64	-4.15	
**AVE[1]**	-1.45	-2.62	-4.01	-5.43
**STD[2]**	0.08	0.09	0.14	0.20
**100mV**	-1.56	-2.75	-4.15	
	-1.56	-2.81	-4.2	
	-1.54	-2.73	-4.11	
	-1.5	-2.92	-4.17	
**AVE**	-1.54	-2.80	-4.16	
**STD**	0.02	0.07	0.03	
**150mV**	-1.64	-2.87	-4.24	-5.73
	-1.54	-2.8	-4.2	-5.67
	-1.44	-2.8	-4.16	-5.57
	-1.5	-2.78	-4.29	-5.6
**AVE**	-1.53	-2.81	-4.22	-5.64
**STD**	0.07	0.03	0.05	0.06
**200mV**	-1.67	-2.94	-4.36	
	-1.65	-2.9	-4.4	
	-1.58	-2.98	-4.49	
	-1.52	-2.78	-4.36	
**AVE**	-1.61	-2.90	-4.40	
**STD**	0.06	0.07	0.05	
**250mV**	-1.83	-3.05	-4.47	
	-1.7	-3.05	-4.43	
	-1.81	-3.14	-4.49	
	-1.84	-3.26	-4.63	
**AVE**	-1.80	-3.13	-4.51	
**STD**	0.06	0.09	0.08	

Supplementary Table 2 | The summary of experimental results for $MAPbBr_{2.15}Cl_{0.85}$ QDs

Conductance / log(G/G_0) \ Bias voltage / mV	P1	P2	P3	P4
**50mV**	-1.52	-2.79	-4.35	
	-1.60	-3.01	-4.58	
	-1.41	-2.65	-4.01	-5.4
**AVE**	-1.51	-2.82	-4.31	
**STD**	0.08	0.15	0.23	
**100mV**	-1.51	-2.81	-4.23	
	-1.56	-2.93	-4.60	
	-1.56	-2.92	-4.33	-5.57
**AVE**	-1.54	-2.89	-4.39	
**STD**	0.02	0.05	0.16	
**150mV**	-1.61	-2.91	-4.34	
	-1.76	-3.09	-4.54	
**AVE**	-1.69	-3.00	-4.44	
**STD**	0.08	0.09	0.10	
**200mV**	-1.65	-2.91	-4.39	
	-1.77	-3.17	-4.70	
**AVE**	-1.71	-3.04	-4.55	
**STD**	0.06	0.13	0.16	
**250mV**	-1.64	-2.92	-4.38	
	-1.84	-3.20	-4.72	
**AVE**	-1.74	-3.06	-4.55	
**ATD**	0.10	0.14	0.17	

1. **AVE = The average of conductance values.**

2. **STD = The standard deviation of conductance values.**

Supplementary Table 3 | The detailed data of DFT calculation

**Schematic**	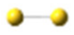	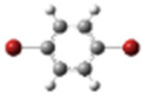	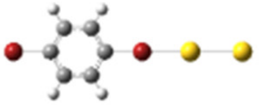	
	Au-Au	1,4-dichlorobenzene	1,4-dichlorobenzene -Au-Au	Au-Cl
**Total energy (HF)**	-270.9484319	-260.9960098	-531.9559787	-0.011537
	-	1,4-diromobenzene	1,4-diromobenzene -Au-Au	Au-Br
**Total energy (HF)**	-	-257.4268114	-528.3907227	-0.0154794
	-	1,4-diiodobenzene	1,4-diiodobenzene -Au-Au	Au-I
**Total energy (HF)**	-	-253.8624627	-524.8314477	-0.0205531
	-	1,4-dithiomethylbenzene	1,4-dithiomethylbenzene -Au-Au	Au-S
**Total energy (HF)**	-	-331.1906692	-602.1798163	-0.0407152

Supplementary Table 4 | The summary of theoretical results

Br-Br distance (Å) / Conductance ($\log(G/G_0)$)	R1	R2	R3	R4	R5	β (Å^{-1})
**8Pb**	5.09	9.85	12.57			
**(Supplementary Figure 26)**	-2.61	-4.46	-2.69			
**12Pb**	4.77	9.55	15.4	18.47		0.54
**(Fig. 4c)**	-3.08	-3.81	-5.52	-3.90		
**16Pb**	4.71	9.41	15.43	20.06	24.21	0.54
**(Supplementary Figure 27)**	-3.11	-3.80	-5.31	-6.67	-5.17	
**16Pb-cross**	5.33	11.43	16.13	20.7		0.72
**(Supplementary Figure 28)**	-4.54	-6.64	-7.89	-6.44		
**16Pb-cross**	7.94	12.71	17.64	21.64		1.2
**(Supplementary Figure 29)**	-5.51	-7.12	-10.70	-7.57		
**18Pb-diagonal**	4.98	10.89	18.2	20.84	20.63	0.87
**(Supplementary Figure 31)**	-2.77	-6.01	-7.86	-8.52	-6.06	
**18Pb**	4.71	9.38	15.39	18.28		0.55
**(Supplementary Figure 36)**	-2.85	-3.63	-5.37	-4.00		
**10Pb**	5.19	9.91	14.94	18.31		0.98
**(Supplementary Figure 37)**	-2.52	-3.72	-6.64	-4.44		
**12Pb-L[a]**	6.76	11.00	16.00	18.78		0.42
**(Supplementary Figure 38)**	-5.51	-6.16	-7.21	-5.67		
**12Pb-L[b]**	10.53	13.7	17.98	19.47		0.88
**(Supplementary Figure 39)**	-5.69	-8.23	-8.67	-7.49		

Supplementary Note 1.

Discussion for Supplementary Figure 1-4.

Supplementary Figure 2 and 4 present the TEM image with the size distribution as the inset and the HRTEM image of spherical $MAPbBr_3$ and $MAPbBr_{2.15}Cl_{0.85}$ QDs. It is observed that typical $MAPbBr_3$ QDs have an average diameter of 3.75 nm with a size deviation of ±1.39 nm (Supplementary Figure 2a) and $MAPbBr_{2.15}Cl_{0.85}$ QDs have an average diameter of 6.32 nm with a deviation of ±1.63 nm (Supplementary Figure 3a). According to the HRTEM image (Supplementary Figure 2b and 3b), interplanar distances corresponding to the (211) crystal plane are 0.243 nm for $MAPbBr_3$ QDs and 0.265 nm for $MAPbBr_{2.15}Cl_{0.85}$ QDs, respectively. The selected area electron diffraction (SAED) pattern also confirms that $MAPbBr_3$ and $MAPbBr_{2.15}Cl_{0.85}$ QDs are high-quality single crystalline with measured *d*-spacing to plane (211). Supplementary Figure 3 presents with the size distribution as the inset and the HRTEM image of spherical $MAPbBr_3$ QDs, which are obtained from the centrifugal speeds of 10000 rpm and 5000 rpm, respectively.

Supplementary Note 2.

Discussion for Supplementary Figure 5.

Photoluminescence (PL), transmission electron microscope (TEM) and energy dispersive spectrometer (EDS) analysis of $MAPbBr_3$ and $MAPbBr_{2.15}Cl_{0.85}$ QDs are further conducted to evaluate the composition of these QDs. In order to synthesize the $MAPbBr_{2.15}Cl_{0.85}$ QDs, we mix the 4:5 molar ratio of MACl and $PbBr_2$. Therefore, the composition of $MAPbBr_{2.15}Cl_{0.85}$ QDs should be approximately $MAPbBr_{2.15}Cl_{0.85}$, which will enlarge the bandgap of QDs and result in the blue shift of PL emission peak of $MAPbBr_{2.15}Cl_{0.85}$ QDs compared to $MAPbBr_3$ QDs. As confirmed in Supplementary Figure 5a, the position of PL emission peak of $MAPbBr_{2.15}Cl_{0.85}$ QDs is at 435 nm (corresponding to 2.85eV) while the position of PL emission peak of $MAPbBr_3$ QDs is at 519 nm (corresponding to 2.39eV). At the same time, we map the $MAPbBr_{2.15}Cl_{0.85}$ QDs with EDS and find in Supplementary Figure 5f that chlorine elements are obviously observed in the $MAPbBr_{2.15}Cl_{0.85}$ QDs.

Supplementary Note 3.

Discussion for Supplementary Figure 6.

In order to confirm the quantum effect of our QDs, we conduct PL measurement and observe a sharp emission peak at 519 nm with an FWHM value of only 24 nm (approximately 105meV) in Supplementary Figure 6a, indicating the superior color saturation than that reported for CdSe or InP-based QDs.[2,3] The UV-vis absorption spectra of $MAPbBr_3$ QDs synthesized by the same method[1] have a band edge of 505 nm indicating a relatively smaller Stokes shift of ~65 meV of PL of $MAPbBr_3$ QDs, implying that the PL emission of QDs originates from direct exciton recombination. In addition, the emission peak is 26 nm (114 meV) blue-shifted compared to that of $MAPbBr_3$ bulk materials.[4] Because of the smaller size of $MAPbBr_3$ QDs relative to the 2-fold exciton Bohr radius (4.0 nm),[5] the observed blue shift of $MAPbBr_3$ QDs can be explained by the quantum confinement effect of QDs, although, the $MAPbBr_3$ QDs synthesized here exhibit less quantum confinement effects than the well-known CdSe- and InP-based QDs.[5]

Time-resolved PL (TRPL) measurements are further performed as shown in Supplementary Figure 6b in order to gain more insight into the exciton recombination dynamics and confirm the quantum confinement of QDs. The PL decay can be described by triexponential fitting, eventually giving an average lifetime of 11ns. Compared to bulk films (average PL lifetime of ~100 ns),[6] the average PL lifetime of colloidal $MAPbPbBr_3$ QDs here is greatly reduced with decreasing size. This suggests that the PL decay of colloidal $MAPbBr_3$ QDs mainly takes place through exciton radiative recombination. This should be another evidence of quantum confinement of QDs, which is also confirmed by Zhang et al[7] and explained by Scholes et al.[8] Therefore, we confirm the $MAPbBr_3$ nanocrystals are QDs based on above results of the Stokes shift and much reduced PL lifetime due to the confinement effect.

Supplementary Note 4.

Discussion for Supplementary Figure 7-8.

The mechanically controllable break junction (MCBJ) is a well-established technique employed to study the atomic and molecular junction since the 1990s,[9-12] and recently has been employed to investigate the charge transport through various nanoscale materials and devices including molecules,[13,14] quantum dots[15] and even assemblies.[16] The schematic of the MCBJ experimental principle is shown in Supplementary Figure 7. The gap spacing of the two gold electrodes can be precisely controlled by the displacement of the pushing rod which is a combination of a stepping motor and a piezo stack. There is a displacement ratio between the displacement of the pushing rod and the moving distance of the nanoscale electrodes, which is typically 10^{-2} for the notched wire sample chips. Since the moving resolution of the piezo stack is 10^{-10} approximately 10^{-11} m, the resolution of the two gold electrodes can reach 10^{-12} approximately 10^{-13} m.[12] Therefore, we can use MCBJ method to precisely control the gap of the created electrodes pair.

Before the MCBJ conductance measurement, an MCBJ chip is prepared as follows: a notched and suspended gold wire (99.99%, 100 μm diameter, Jiaming, Beijing) is fixed by two drops of epoxy glues (Stycast 2850 FT with catalyst 9) on a spring steel sheet (10 mm × 30 mm with 0.2 mm thickness). And the distance between two epoxy glues is required less than 0.5 mm to ensure stability during the conductance measurements. The chip is put into the oven with 60 °C for two hours to solidify the epoxy glues. Then the chip is fixed on the MCBJ setup for the following break junction measurements. The MCBJ setup for the whole view with a suspended system and the enlarged view is shown in Supplementary Figure 8a and 8b.

The pre-cleaned polytef liquid cell and a perfluoroelastomer O-ring (FFKM 6.07 × 1.78, Wuxi Bo Yate Sealing Technology Development Co. Ltd.) are pressed above the chip to prevent liquid leakage. Then the perovskite QDs solution is added into the liquid cell. During the process of MCBJ measurements, the substrate is first fixed by two counter supports at both sides, and then a pushing rod driven by a stepping motor and a piezo stack is employed to bend the substrate in the middle, resulting in the breakage of the notched gold wire at horizontal direction to form a nano-gap. The fractured gold wire will capture a single perovskite QD with halogen anchors and form the Au-QD-Au junction. Owing to the elasticity of the stainless-steel substrate, the gold wire will connect again during the returning process of the pushing rod. The conductance of the single-QD junctions can be

collected after repeating this process thousands of times.

By applying a bias voltage of 100 mV, the current passing through electrode pairs is measured by a lab-built logarithm *I-V* converter with a sampling rate of 20 *kHz*. For each perovskite QD, the breaking/re-connecting process is repeated and recorded for thousands of times dynamically, and the most probable conductance of an Au-QD-Au junction is obtained by statistical analysis of the data.

Supplementary Note 5.

Discussion for Supplementary Figure 9.

The impurity of the solvent and the stretching rate of gold atomic chain will play a significant role in experimental results. It is reported that the solution environment is considered as a key factor to determine the stability of perovskite QDs.[17] The polar solvents will induce photoluminescence quantum quenching or even destroy the QDs. The non-polar solvent, 1,3,5-trimethylbenzene (TMB), has high solubility for perovskite QDs. To exclude the influence of solvent, we use TMB without target molecules as a blank experiment for the calibration of distance for MCBJ experiments.[18,19] The conductance histogram and 2D conductance versus relative distance (Δz) histogram are shown in Supplementary Figure 9a and 9b. The histogram has no clear conductance plateau and expresses an exponential decay after the rupture of gold-gold atomic contact at quantum conductance G_0, which means that the solvent is pure and clean.

To determine the calibration of the distance measurements, we correct the pizeo stretching rate and the snap-back distance. Since there is a conductance of G_0 in the linear gold atomic chain, we assume that $\log(G/G_0) = -\alpha z$. z=0 represented the distance between the terminating gold atoms which is equal to the equilibrium gold-gold separation. Since the separation we measured is Δz=z-Δz_{corr}, where Δz_{corr} represents the snap-back distance of gold atoms, a plot of $\log(G/G_0)$ versus Δz has a slope of $-\alpha$ and an intercept of αz_{corr}. From the conductance distribution of the most probable intercepts and slopes, we obtain the most probable values of α and Δz_{corr}. According to the tunneling decay constant ($\log[\Delta G/G_0]/\Delta z$ = -5.5 nm^{-1}) reported by previous literature,[20,13] the relative displacement distribution in the conductance ranged from $10^{-3.5}$ G_0 to $10^{-5.5}$ G_0 is calibrated at 0.36 nm (-2/-5.5= approximately 0.36), as shown in Supplementary Figure 9c. By calibrating the relative distance distribution from $10^{-3.5}$ G_0 to $10^{-5.5}$ G_0 to 0.36 nm, the stretching rate is determined and can be used for further statistical analysis of the 2D conductance histograms and the relative displacement distributions of the molecular junctions.

After calibrating the stretching rate of the gold atomic chain, we can carry out the single-molecule conductance measurement of perovskite QDs using MCBJ technique. For $MAPbBr_3$ and $MAPbBr_{2.15}Cl_{0.85}$ perovskite QDs in this paper, the displacement distribution of the Δz can be determined based on the corrected stretching rate used in the above method, which can reflect the gap spacing and the lattice distance of the adjacent Br atoms precisely. According to the distribution of

the conductance peaks in Fig. 3c, the conductance statistical ranges of the three conductance plateaus should be 10^{-1} to 10^{-2} G_0, 10^{-2} to $10^{-3.2}$ G_0, $10^{-3.2}$ to $10^{-4.8}$ G_0 for $MAPbBr_3$ and 10^{-1} to 10^{-2} G_0, 10^{-2} to $10^{-3.4}$ G_0, $10^{-3.4}$ to $10^{-4.9}$ G_0 for $MAPbBr_{2.15}Cl_{0.85}$ perovskite QDs, respectively. Therefore, the relative displacement distributions are determined to be 0.18±0.16 nm, 0.64±0.23 nm, 1.17±0.28 nm for $MAPbBr_3$, and 0.18±0.23 nm, 0.63±0.21 nm, 1.19±0.27 nm for $MAPbBr_{2.15}Cl_{0.85}$ perovskite QDs respectively. The average displacement differences are approximately 0.5nm, which is quite in accordance with the adjacent distances of Br atom. In our experiment, the distance is determined directly from tunneling current other than the piezo traveling distance or the reduction ratio from sample to sample, thus the variation in the mechanical reduction ratio will not influence the calibration of the distance measurements as we have discussed.

Supplementary Note 6.

Discussion for Supplementary Figure 10.

The ligands of perovskite QDs are recognized to enhance colloidal stability and suppress the QDs aggregation effects.[21] To exclude the effects from the ligands and synthetic raw materials (oleic acid, octylamine, $PbCl_2$, $PbBr_2$, MACl and MABr) during the MCBJ measurement of perovskite QDs, the control experiments of all the ligands are carried out in the solvent, γ-butyrolactone, and also use the ultrasonic concussion for at least 30 minutes to guarantee the solubility.[22,23] The 1D conductance histogram and 2D conductance-distance histogram are shown in Supplementary Figure 10. The obvious conductance plateau can be observed in $PbBr_2$, while no clear conductance signal can be observed in other ligands and raw materials, suggesting that the conductance signal only derives from Au-Br interaction. The $PbBr_2$ would change to $(PbBr_6)^{4-}$ and $(Pb_2Br_9)^{5-}$ in the solvent of γ-butyrolactone, corresponding to the two conductance peaks in Supplementary Figure 10g.[24-26]

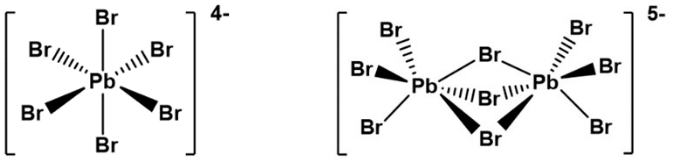

Supplementary Note 7.

Discussion for Supplementary Figure 11.

We also study the current-voltage characteristics of $MAPbBr_3$ and $MAPbBr_{2.15}Cl_{0.85}$ perovskite QDs. It is found that the current gradually increase and the current-voltage curves express a traditional linear relation, which corresponds well with the Simmons model of *I-V* characteristics at low bias voltage, suggesting the charge transport accords with a typical off-resonant coherent tunneling mechanism.

Supplementary Note 8.

Discussion for Supplementray Figure 12.

We further investigate the charge transport properties of $MAPbBr_3$ and $MAPbBr_{2.15}Cl_{0.85}$ perovskite QDs at high bias voltage (300 mV and 400 mV). It is found that the single-QD junctions become quite unstable at high bias voltage and the conductance values of three plateaus are difficult to identify, which may be due to the destruction of the perovskite clusters at such high electric fields.

Supplementary Note 9.

Discussion for Supplementary Figure 14.

Due to the complicated and changeable patterns of jump conductance traces of perovskite QDs, the fourth "jump conductance" is hard to see in traditional 2D conductance vs displacement histograms. To clearly display the "jump conductance", we carry out a new method referring to the treatment of 2D relative force vs relative displacement histograms using conductive AFM technique.[27] The jump points of the fourth step of single molecular conductance vs displacement traces are determined as follows:

Every conductance trace which is composed of over 5000 data points will be selected for processing. Firstly, we select the region ranged from the jump point to the next 250 points (approximately 0.1 nm) for pretreating analysis. We set a threshold value of the conductance variation to identify the jump points of the traces, shown in Supplementary Figure 14a. The threshold value of conductance should be set in a reasonable range depending on the features of the jump molecular plateaus and the corresponding locations because the statistical numbers of selected traces will decrease if this threshold value is too large and many non-jump traces will be selected if the value is too small. In this case, we determine the jump point if the conductance value of this data point is 0.4 $\log(G/G_0)$ smaller than the next 30 data points, which included more than 70% of all the conductance traces.

Then we set the origin of the displacement axis (x-axis) and conductance axis (y-axis) at the 150 data points (approximately 0.06nm) before the jump points for every selected trace. The relative displacement and relative conductance are used as new x-axis and y-axis to clearly display the jump location of the fourth conductance. All the traces are processed in the same procedures using the automated algorithm mentioned above to obtain the new 2D relative conductance (G) vs displacement (Δz) histograms, as shown in Fig. 2d. The peak of a Gaussian fit (blue curves in Fig. 2d) towards the distribution of conductance is also added for every displacement bin to display the conductance profile by statistics. To quantify the variation of the "jump conductance", we calculate the relative height of the "jump conductance" considering the jump points as a reference with the relative displacement ranged from the starting jump point to the next highest jump point. As shown in Supplementary Figure 14b and S14c, the most probable relative G of the fourth steps is approximately 0.81 log (G/G_0) and the "jump conductance" is approximately -4.31 log (G/G_0).

Supplementary Note 10.

Discussion for Supplementary Figure 15.

We also carry out the same procedures using the automated algorithm to display the "jump curves" of $MAPbBr_{2.15}Cl_{0.85}$ perovskite QDs. As shown in Supplementary Figure 15, the 2D relative conductance (G) vs displacement (Δz) histogram expresses a clear "jump conductance", which includes over approximately 60% of all the conductance-distance curves. The 1D conductance distribution histograms (Supplementary Figure 15a and Supplementary Figure 15b) show that the most probable relative G of the fourth steps is approximately 0.75 log (G/G_0) and the "jump conductance" is approximately -4.25 log (G/G_0), which is quite similar with $MAPbBr_3$ perovskite QDs.

Supplementary Note 11.

Discussion for Fig. 3f.

To further prove that it is the Br atoms anchoring to the gold electrodes during the single-molecule break junction measurement, we use the shell-isolated nanoparticle-enhanced Raman spectroscopy (SHINERS) technique to detect the Au-Br interaction on the gold substrates with perovskite QDs. 3.65 mM perovskite QDs are dropped on the evaporated gold films and then the as-prepared shell-isolated gold nanoparticles[17] are added for Raman enhancement. The Raman spectroscopy is collected using a laser of 633nm with optical grate of 1800, hole of 300 and power of approximate 3.94 $mW/\mu m^2$. As shown in Fig. 3f, two distinct Raman peaks can be observed at approximately 180 cm^{-1} in $MAPbBr_3$ and $MAPbBr_{2.15}Cl_{0.85}$ perovskite QDs, which belongs to typical peak of the Au-Br bond.[28,29]

Supplementary Note 12.

Discussion for Supplementary Figure 16.

In order to provide further evidence that the Au-I interaction cannot form the stable single-QD junction, we have also measured the electrical properties of $MAPbBr_{2.15}I_{0.85}$ perovskite QDs. The experimental results show that neither the peak of gold-gold atomic junction nor the conductance plateaus of the single-QD junction can be observed in $MAPbBr_{2.15}I_{0.85}$ QDs, which is similar to the charge transport properties of $MAPbBrI_3$ QDs. Therefore, it can be demonstrated that the Au-I interaction cannot form stable single-QD junctions due to its strong bond energy and poorer stability of the crystal structure.

Supplementary Note 13.

Discussion for Supplementary Figure 17.

In order to prove that the sizes and diameters of QDs have no impact on their electrical properties, we carry out the MCBJ measurements using the perovskite quantum dots obtained with the centrifugal speeds of 5000 rpm and 10000 rpm (as shown in Supplementary Figure 3). The $MAPbBr_3$ QDs centrifuged with different centrifugal speeds express similar conductance values located at $10^{-1.54}$, $10^{-2.80}$ and $10^{-4.32}$ for 5000 rpm, $10^{-1.43}$, $10^{-2.72}$ and $10^{-4.28}$ for 10000 rpm, respectively, and the difference of adjacent statistical lengths matches well with the adjacent lattice distance of Br, which prove that the conductance plateaus we measured originate from the perovskite crystal cells rather than the entire perovskite QDs.

Supplementary Note 14.

Discussion for Supplementary Figure 18-19.

In order to confirm that the experimental data we measured has high repeatability and quality, we repeated the MCBJ measurements of $MAPbBr_3$ QDs several times at different bias voltages. The experimental data presents that all 1D conductance histograms display three or four clear conductance plateaus with small standard deviations, suggesting that the experimental data is highly reproducible and robust.

Supplementary Note 15.

Discussion for Supplementary Figure 20-22.

The spectral clustering algorithm is a state of art clustering technique which provides a partition of data and assigns similar data traces into clusters. Here we firstly review the spectral clustering algorithm according to the Ng *et al.* on the 1D conductance histograms.[30]

Given histogram data $H = \{h_1, h_2, ..., h_M\}$ in R^N (dividing the conductance axis to discrete N bins) that we want to cluster into K clusters:

1. Form the affinity matrix $A \in R^{M\times M}$ defined by $A_{ij} = C_{ij} + 1$ if $i \neq j$, and $A_{ii} = 0$.
2. Define D to be the diagonal matrix whose (i, j)-element is the sum of A's i-th row, and construct the matrix $L=D^{-1/2}AD^{-1/2} - I$.
3. Find $x_1, x_2, ... x_K$, the K largest eigenvectors of L, and form the matrix $X = [x_1, x_2, ... x_K]$ belong to $R^{M\times K}$ by stacking the eigenvectors in columns.
4. Treating each row of X as a point in R^K, cluster them into K clusters via K-means++.
5. Finally, assign the original points h_i to cluster j if and only if row i of the matrix X was assigned to cluster j.

Here we construct the affinity matrix A specified different from the usual one (the Gaussian (aka RBF) kernel), the other steps are almost the same as described in Ng *et a*l.[30] Here we define the C_{ij} as the cross-correlation between histogram h_i and h_j as follow:

$$C_{ij} = \frac{\langle[h_i-\langle h_i\rangle][[h_j-\langle h_j\rangle]\rangle}{\sqrt{\langle[h_i-\langle h_i\rangle]^2\rangle\langle[h_j-\langle h_j\rangle]^2\rangle}} \qquad (1)$$

where $\langle h_i\rangle$ represents the average value of histogram h_i, the values of C range from [-1, 1], so we add one to make the elements of affinity matrix A nonnegative to meet the spectral clustering requirements. h_i is the conductance histogram for the i-th individual trace, M is the number of conductance traces, N is the number of the histogram bins.

Supplementary Note 16.

Discussion for Supplementary Table 3.

To calculate the gold-halogen binding energy, we use typical molecules (1,4-dichlorobenzene, 1,4-bromobenzene, 1,4-diiodobenzene) containing halogen as models. A gold dimer is used to bond to halogen following an example shown in *J. Am. Chem. Soc.* **133**, 2136-2139 (2011). All of the structures are optimized using DFT with a B3LYP density functional with 6-311++(d,f) to describe C, H, S, Lanl2DZ and the effective core potential of Au, Cl, Br and I. The binding energy of the Au-molecule complex is E_T(complex) – [E_T(molecule) + E_T(Au-Au)], where E_T(complex), E_T(molecule) and E_T(Au-Au) are the total energies of the complex, the molecule and the dimer respectively. As a reference, we also calculate the bond energy of an Au-S coordination bond by substituting halogen atoms on the molecule for thiomethyl. Detailed data are shown in Supplemental Table 3.

The bonding energy of Au-Cl, Au-Br, Au-I, Au-S, namely HF(Au-X) × 2625.5 kJ / mol are 30.29, 40.64, 53.96, 106.89 kJ / mol respectively. DFT calculation shows that the Au-halogen binding energy accords with the order of Au-I > Au-Br > Au-Cl. The bonding energy Au-Br is lower than that of the Au-S bond,[31,32] which is consistent with the sliding through different lattices on the QD via the Au-Br interaction.

Supplementary References

1. Zhang, F., *et al.* Brightly luminescent and color-tunable colloidal $CH_3NH_3PbX_3$ (X= Br, I, Cl) quantum dots: potential alternatives for display technology. *ACS nano* **9**, 4533-4542 (2015).
2. Kovalenko, M.V., *et al.* Prospects of nanoscience with nanocrystals. *ACS Nano* **9**, 1012-1057 (2018).
3. Pradhan, N., Reifsnyder, D., Xie, R., Aldana, J. & Peng, X. Surface ligand dynamics in growth of nanocrystals. *J. Am. Chem. Soc.* **129**, 9500-9509 (2007).
4. Kojima, A., Ikegami, M., Teshima, K. & Miyasaka, T. Highly luminescent lead bromide perovskite nanoparticles synthesized with porous alumina media. *Chem. Lett.* **41**, 397-399 (2012).
5. Tanaka, K., *et al.* Comparative study on the excitons in lead-halide-based perovskite-type crystals $CH_3NH_3PbBr_3$ $CH_3NH_3PbI_3$. *Solid State Commun.* **127**, 619-623 (2003).
6. Zhang, M., *et al.* Composition-dependent photoluminescence intensity and prolonged recombination lifetime of perovskite $CH_3NH_3PbBr_{3-x}Cl_x$ films. *Chem. Commun.* **50**, 11727-11730 (2014).
7. Scholes, G.D. & Rumbles, G. Excitons in nanoscale systems. *Nat. Mater.* **5**, 683-696 (2006).
8. Yanson, A., Bollinger, G.R., Van den Brom, H., Agrait, N. & Van Ruitenbeek, J. Formation and manipulation of a metallic wire of single gold atoms. *Nature* **395**, 783-785 (1998).
9. Reed, M.A., Zhou, C., Muller, C., Burgin, T. & Tour, J. Conductance of a molecular junction. *Science* **278**, 252-254 (1997).
10. Muller, C., Van Ruitenbeek, J. & De Jongh, L. Conductance and supercurrent discontinuities in atomic-scale metallic constrictions of variable width. *Phys. Rev. Lett.* **69**, 140 (1992).
11. Muller, C., Van Ruitenbeek, J. & De Jongh, L. Experimental observation of the transition from weak link to tunnel junction. *Physica C* **191**, 485-504 (1992).
12. Schwarz, F., *et al.* Field-induced conductance switching by charge-state alternation in organometallic single-molecule junctions. *Nat. Nanotechnol.* **11**, 170-176 (2016).
13. Lumbroso, O.S., Simine, L., Nitzan, A., Segal, D. & Tal, O. Electronic noise due to temperature differences in atomic-scale junctions. *Nature* **562**, 240-244 (2018).
14. Lovat, G., *et al.* Room-temperature current blockade in atomically defined single-cluster junctions. *Nat. Nanotechnol.* **12**, 1050-1054 (2017).
15. Frisenda, R., Janssen, V.A., Grozema, F.C., van der Zant, H.S. & Renaud, N. Mechanically

controlled quantum interference in individual π-stacked dimers. *Nat. Chem.* **8**, 1099-1104 (2016).

16. Capozzi, B., *et al.* Single-molecule diodes with high rectification ratios through environmental control. *Nat. Nanotechnol.* **10**, 522-528 (2015).
17. Hong, W.J., *et al.* Single molecular conductance of tolanes: experimental and theoretical study on the junction evolution dependent on the anchoring group. *J. Am. Chem. Soc.* **134**, 2292-2304 (2012).
18. Fatemi, V., Kamenetska, M., Neaton, J.B. & Venkataraman, L. Environmental control of single-molecule junction transport. *Nano Lett.* **11**, 1988-1992 (2011).
19. Hong, W., *et al.* An MCBJ case study: The influence of π-conjugation on the single-molecule conductance at a solid/liquid interface. *Beilstein J. Nanotechnol.* **2**, 699-713 (2011).
20. Pan, A., *et al.* Insight into the ligand-mediated synthesis of colloidal $CsPbBr_3$ perovskite nanocrystals: The role of organic acid, base, and cesium precursors. *ACS Nano* **10**, 7943-7954 (2016).
21. Saidaminov, M.I., Abdelhady, A.L., Maculan, G. & Bakr, O.M. Retrograde solubility of formamidinium and methylammonium lead halide perovskites enabling rapid single crystal growth. *Chem. Commun.* **51**, 17658-17661 (2015).
22. Clever, H.L. & Johnston, F.J. The solubility of some sparingly soluble lead salts: an evaluation of the solubility in water and aqueous electrolyte solution. *J. Phys. Chem. Ref. Data* **9**, 751-784 (1980).
23. Bohun, A., Dolejší, J. & Barta, Č. The absorption and luminescence of $(PbCl_6)^{4-}$ and $(PbBr_6)^{4-}$ complexes. *J. Phys. B* **20**, 803-807 (1970).
24. Nergararian, A. & Berka, L. Anomalous absorption bands in ultraviolet spectra of halide solutions. *Anal. Chem.* **44**, 2192-2195 (1972).
25. Mousdis, G.A., Gionis, V., Papavassiliou, G.C., Raptopoulou, C. & Terzis, A. Preparation, structure and optical properties of $[CH_3SC(=NH_2)NH_2]_3PbI_5$,$[CH_3SC(=NH_2)NH_2]_4Pb_2Br_8$ and $[CH_3SC(=NH_2)NH_2]_3PbCl_5 \cdot CH_3SC(=NH_2)NH_2Cl$. *J. Mater. Chem.* **8**, 2259-2262 (1998).
26. Frei, M., Aradhya, S.V., Koentopp, M., Hybertsen, M.S. & Venkataraman, L. Mechanics and chemistry: single molecule bond rupture forces correlate with molecular backbone structure. *Nano Lett.* **11**, 1518-1523 (2011).
27. Peng, Y., Niu, Z., Huang, W., Chen, S. & Li, Z. Surface-enhanced Raman scattering studies of 1,

10-phenanthroline adsorption and its surface complexes on a gold electrode. *J. Phys. Chem. B* **109**, 10880-10885 (2005).

28. Gao, P., Patterson, M.L., Tadayyoni, M. & Weaver, M.J. Gold as a ubiquitous substrate for intense surface-enhanced Raman scattering. *Langmuir* **1**, 173-176 (1985).
29. Ng, A.Y., Jordan, M.I. & Weiss, Y. On spectral clustering: Analysis and an algorithm. in *Advances in neural information processing systems* 849-856 (2002).
30. Bruot, C., Hihath, J. & Tao, N. Mechanically controlled molecular orbital alignment in single molecule junctions. *Nat. Nanotechnol.* **7**, 35-40 (2012).
31. Park, Y.S., *et al.* Contact chemistry and single-molecule conductance: a comparison of phosphines, methyl sulfides, and amines. *J. Am. Chem. Soc.* **129**, 15768-15769 (2007).
32. Giorgi, G., Yoshihara, T. & Yamashita, K. Structural and electronic features of small hybrid organic–inorganic halide perovskite clusters: a theoretical analysis. *Phys. Chem. Chem. Phys.* **18**, 27124-27132 (2016).
